# A *Slc5a6*-deficient mouse model reveals metabolically driven cardiomyopathy with therapeutic potential for vitamin-based intervention

**DOI:** 10.1172/jci.insight.200381

**Published:** 2026-06-02

**Authors:** Millie O. Fullerton, Lauren C. Phillips, Rachael E. Redgrave, Luke Spray, Vincent Haufroid, George Merces, Scott T. Kerridge, Gavin D. Richardson, Nathalie Mercier, Dominique Roland, Rebecca Crossley, Andrew D.H. Morgan, Joseph P. Dewulf, John Burn, Simon D. Bamforth, Helen M. Phillips

**Affiliations:** 1 Biosciences Institute, Newcastle University, Newcastle upon Tyne, United Kingdom.; 2 Department of Pharmacology, University of Oxford, United Kingdom.; 3 Translational and Clinical Research Institute, Newcastle University, Newcastle upon Tyne, United Kingdom.; 4 Louvain Center for Toxicology and Applied Pharmacology (LTAP), Institut de Recherche Expérimentale et Clinique (IREC), UCLouvain, Brussels, Belgium.; 5 Image Analysis Unit, Newcastle University, Newcastle upon Tyne, United Kingdom.; 6 Department of Pediatrics, CHU HELORA Jo, La Louvière, Belgium.; 7 Department of Human Genetics, Center for Inherited Metabolic Disorders, Institute of Pathology and Genetics, Gosselies, Belgium.; 8 Metabolic Research Group, de Duve Institute, UCLouvain, Brussels, Belgium.; 9 Biochemical Genetics and Newborn Screening Laboratory, Department of laboratory medicine & Institut des Maladies Rares, Cliniques Universitaires Saint-Luc, UCLouvain, Brussels, Belgium.

**Keywords:** Cardiology, Metabolism, Cardiovascular disease, Mouse models

## Abstract

The sodium-dependent multivitamin transporter, encoded by *SLC5A6*, mediates cellular uptake of biotin and pantothenic acid, essential cofactors for energy metabolism. We identified 2 families with *SLC5A6* mutations presenting with early-onset dilated cardiomyopathy (DCM). To investigate the link between vitamin deficiency and cardiomyopathy, we generated a cardiac-specific *SLC5A6*-knockout (*Slc5a6^cKO^*) mouse model and evaluated the impact of vitamin supplementation. *Slc5a6^cKO^* mice developed progressive cardiac dysfunction, culminating in cardiac pathology and premature death at 26 weeks; earlier stages exhibited cardiomyocyte hypertrophy, fibrosis, impaired coenzyme A synthesis, and metabolic imbalance, indicating progression toward cardiomyopathy. Cardiac magnetic resonance imaging and ECG confirmed progressive functional decline. Proteomic analysis revealed early mitochondrial metabolic disruption and extracellular matrix protein upregulation at 8 weeks, preceding overt cardiac dysfunction. Strikingly, vitamin supplementation from preconception onwards prevented the cardiac phenotype, preserving cardiac structure, function, morphology and survival. This paralleled the clinical outcome in one patient who received early vitamin treatment, compared with another who required a heart transplant without vitamin treatment. This study establishes a direct link between *SLC5A6*-mediated vitamin transport, mitochondrial function, and cardiac health. It highlights how vitamin deficiency contributes to cardiomyopathy pathogenesis and supports early vitamin supplementation as a potential therapeutic strategy for metabolic cardiomyopathies.

## Abstract 2

Rare gene defects that block vitamin uptake can damage the heart, but early treatment with simple vitamin supplements can prevent disease and protect heart function.

## Graphical abstract

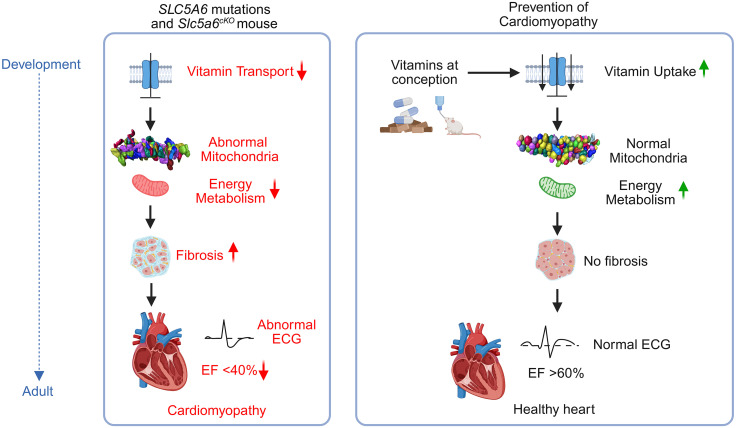

## Introduction

Dilated cardiomyopathy (DCM) is a leading cause of cardiovascular morbidity and mortality, affecting approximately 1 in 250 people in the United Kingdom, characterized by enlargement and dilation of one or both ventricles with impaired contractility, reduced left ventricular ejection fraction (EF), and heart failure. Pediatric cases of DCM are particularly severe, with over one-third of affected children requiring heart transplantation (1). The etiology of DCM includes both genetic and environmental factors, such as mutations in sarcomeric genes (2) or exposure to cardiotoxic drugs (3). Many cases of DCM, however, are idiopathic, and the complex pathological mechanisms underlying DCM are still not well understood. The heart is the most metabolically demanding organ in the body, requiring a high ATP turnover in cardiomyocytes; given the critical role of mitochondria in energy metabolism, disruptions in mitochondrial dynamics and function have been strongly linked to cardiovascular disease and heart failure (4).

*SLC5A6* encodes the highly conserved and ubiquitously expressed sodium-dependent multivitamin transporter (SMVT), a member of the SLC5 family within the solute carrier (SLC) superfamily. These sodium/solute symporters allow the unilateral transport of molecules across the cell membrane (5, 6). SMVT is required for the uptake of water-soluble vitamins, biotin (vitamin B7), pantothenic acid (vitamin B5), and lipoic acid (5–7). Biotin and pantothenic acid are acquired from the diet (5) and are vital for energy production via the tricarboxylic acid (TCA) cycle, amino acid catabolism, and fatty acid synthesis.

Patients with homozygous or compound heterozygous mutations in *SLC5A6* encompass an autosomal recessive disorder and exhibit a wide spectrum of multisystemic clinical manifestations, including developmental and growth delay, neurodegenerative disorders, gastrointestinal problems, immunodeficiency, seizures, and osteopenia (8–20). The clinical symptoms in patients with *SLC5A6* mutations typically manifest between 1 week and 15 years, with 18% of patients dying with no reported treatment. It is an autosomal recessive disease, but with no obvious genotype-phenotype correlation. A third of patients exhibit cardiac complications, including ventricular fibrillation (10), left ventricle dysfunction (15), non-specific ST and T wave abnormalities (9, 19), and DCM, requiring heart transplantation (11, 14). However, the role of *SLC5A6* in cardiovascular health and function has not been studied.

Here, we describe 3 patients, 2 of whom are from a newly identified consanguineous family, and compare them with a previously published patient (15), all of whom presented with early-onset DCM and homozygous *SLC5A6* mutations. Family 1 has 2 affected siblings, one of whom died prematurely, while the other had a life-saving heart transplant, prior to receiving vitamin supplementation. In the second family, the patient similarly presented with severe heart failure but had a successful response to early vitamin supplementation, thus preventing the need for a transplant.

To investigate a specific association between cardiomyopathy and vitamin deficiency, we generated a cardiac-specific conditional deletion of *Slc5a6* in mice (*Slc5a6^cKO^*). These mice exhibited a progressive cardiomyopathy phenotype, culminating in premature death around 26 weeks. We identified abnormalities in cardiac function and protein expression preceding the onset of histological manifestations. Metabolomic and proteomic analyses of *Slc5a6^cKO^* hearts revealed disruptions in metabolic pathways and an increase in fibrosis-associated proteins from as early as 8 weeks of age. Remarkably, vitamin supplementation completely ameliorated the cardiomyopathy phenotype, with functional, pathological, and protein expression changes completely absent.

## Results

### Clinical manifestations and progression of DCM in 2 consanguineous families.

In Family 1, which is of Pakistani ethnicity, 3 siblings were born to healthy consanguineous parents (Figure 1A). All pregnancies carried to full term without any complications. Child II-1 died at 24 months due to DCM but no clinical details were available. Patient II-2 developed an acute illness at 9 months, characterized by hypoglycemia and lethargy, which rapidly progressed to respiratory failure, necessitating pediatric intensive care unit admission and mechanical ventilation for 2 weeks. During this time, total parenteral nutrition (TPN) was given, and a clinical diagnosis of DCM was entered in the patient’s clinical notes, although no objective measures of cardiac dilation were available. A subsequent admission occurred at 21 months due to cardiac dysfunction requiring inotropic support, and the patient developed pneumonia, left-sided paralysis, and seizures. Mild developmental delay was noted, and nutritional support was administered via nasogastric tube due to feeding difficulties. The patient then remained well for 2 years but relapsed at 4 years of age. Chest x-ray showed an enlarged dilated heart in Patient II-2, with a cardiothoracic ratio (CTR) of 0.57 (Figure 1B), consistent with cardiomegaly (21). Echocardiogram demonstrated globally impaired ventricular function with a left ventricular fractional shortening of only 5%, indicative of severe dysfunction. A left ventricular assist device (LVAD) was inserted and over the next 7 weeks repeated echocardiography demonstrated apparent improvement in left ventricular function. The patient was given vitamin supplementation of riboflavin, thiamine, ascorbic acid, and ubiquinone during this time. At 5 years old, another acute cardiac event triggered readmission with a second LVAD inserted for 2 weeks and TPN was administered. Cardiac function recovered and was stable for 2 years. At this point, the patient presented with lethargy and cardiorespiratory insufficiency and a third LVAD was inserted until a donor heart was transplanted 1 week later. Clinical follow-up showed no organ rejection and good cardiac function, and the stability of the transplant is maintained on tacrolimus 1 mg daily and mycophenolate mofetil 500 mg twice daily. Additional clinical complications included developmental delay, persistent left hemiplegia, complete areflexia, and recurrent seizures. Metabolic screening at 7 years demonstrated increased excretion of 3-hydroxyisovaleric acid (3-HIA) in urine, and increased 3-hydroxyisovaleryl-carnitine (C5OH) in both urine and a blood spot, consistent with 3-methylcrotonyl-Coenzyme A carboxylase (MCC) and biotinidase deficiency (22). At 14 years, based on the molecular findings (described below), supplementation was commenced with daily doses of pantothenic acid (550 mg), lipoic acid (400 mg), and biotin (10 mg). Subsequently daily ferrous sulphate (210 mg) was added, 4 years later, after diagnosis of iron deficiency associated with recurrent episodes of loose stools and hair loss. Epilepsy is controlled with twice daily zonesamide (200 mg), lamotrigine (225 mg), clonazepam (10 mg), and brivaracetam (50 mg). Infection is suppressed with 480 mg co-trimoxazole daily. Assessing the impact of the vitamin supplements has been challenging in an adolescent with multiple medical problems. The parents are excellent observers and consider growth, development, and epilepsy control to have all improved after 6 years on supplements. Puberty commenced normally. She continues to have occasional seizures coinciding with menstruation.

A myocardial biopsy obtained during LVAD insertion, stained by H&E, showed myocardial disarray, featuring hypertrophied cardiomyocytes (Figure 1C) with large, irregular, hyperchromatic nuclei. Pale stained cells correspond to myofibrillar loss within cardiomyocytes (Figure 1D). Transmission electron microscopy (TEM) images of the left ventricle revealed sarcomeric wasting and disarray. In some areas, the sarcomeres had fragmented, creating white patches, and the organized parallel alignment of the sarcomeres was lost. Z band disarray was evident, indicated by variations in the thickness of the lines. Additionally, large clusters of mitochondria were observed, with signs of fragmentation (Figure 1, E–H). The examining pathologist diagnosed cardiomyopathy with no morphological evidence as to the cause.

In Family 2 (15), consanguineous parents from Tunisia, had 5 children (Figure 1I). Child II-2 died of unexplained multisystemic decompensation and severe cardiac dysfunction at 8 months (genetic testing was not available at that time). Patient II-5 presented with DCM with severe cardiac decompensation at 5 months, with a left ventricular EF of 32% (normal > 60%; classed as day 0 in Figure 1J); however, further echocardiographic parameters or cardiac dimensional measurements could not be obtained. Treatment with cardiac drugs (adrenaline, milrinone, and furosemide) for 3 days resulted in EF improvement to 62% by day 10. However, by day 22, the patient had relapsed with an EF of 17%, necessitating intensive cardiac support with an increasing number of cardiac drugs (digoxin, furosemide, spironolactone, carvedilol, and lisinopril). Additionally, following an abnormal plasma acylcarnitine profile and a persistent increase in 3-HIA in the urine, biotin supplementation (10 mg on day 22, increased to 15 mg on day 33), and then pantothenic acid (100 mg from day 64) were initiated. Following the increase in biotin dose to 15 mg, the EF increased to 45% and the patient was maintained on vitamins and 4 cardiac drugs (digoxin, furosemide, carvedilol, and lisinopril) for a further 79 days. The number of cardiac drugs were slowly reduced as the EF rose to a healthy 66% on day 121 and 75% on day 150 (Figure 1J). Bivitamin supplementation remains ongoing, and the EF has remained within the normal range. The patient also presented with severe hypogammaglobulinemia that interestingly corrected itself after vitamin supplementation.

### Pathogenic missense mutations in SLC5A6.

Whole-exome sequencing of Family 1 revealed a homozygous missense variant in *SLC5A6* ([GRCh37] Chr2:27427777 C>T) in Patients II-1 and II-2. The mutation results in a substitution of arginine at position 253 to a tryptophan (p.R253W). All unaffected family members were heterozygous for the variant (Figure 1A). The mutation, in exon 9 of *SLC5A6*, has an allele frequency of 0.00001983 (gnomADv4.1) and a high pathogenicity prediction (CADD score: 27.4).

Both the p.R253W and p.P437L mutation (C>T) in *SLC5A6* previously reported in Family 2 (15) affect highly conserved amino acids (Figure 1K). Within the SMVT protein **(**, the p.R253W mutation is located on the extracellular loop between transmembrane domains 6 and 7, spanning the hinge region of the LeuT fold and p.P437L is within transmembrane domain 11 (Figure 1L). In silico protein modeling indicated that the substitution of arginine to tryptophan in p.R253W introduces a large bulky aromatic ring, which is predicted to induce steric hindrance and prevent the formation of a salt bridge with E91 (Supplemental Figure 1, A–E; supplemental material available online with this article; https://doi.org/10.1172/jci.insight.200381DS1). Similarly, P437L repositions the large intracellular loop and a polar bond is formed between L437 and V434 (Supplemental Figure 1, F–J). Therefore, structural modeling predicts that both of these pathogenic mutations in *SLC5A6* may induce conformational changes in the tertiary structure of the SLC5A6 protein, but further experimental validation of the functional impact is required in future studies.

### Slc5a6^cKO^ mice develop cardiomyopathy.

We confirmed that SLC5A6 was expressed in the mouse and human embryonic heart (Supplemental Figure 2, A–G). Initially, it was found that mice constitutively deficient for *Slc5a6* (Supplemental Figure 2H) exhibited embryonic lethality, with underdeveloped embryos at embryonic day 9.5 (E9.5) and E10.5 (Supplemental Figure 2, I–L). The number of *Slc5a6^Tm1a/Tm1a^* mutant embryos was significantly underrepresented when collected between E9.5 and E15.5 (Supplemental Figure 2M).

A cardiac-specific *Slc5a6*-knockout (*Slc5a6^cKO^*) mouse line was then produced by crossing conditional *Slc5a6^Tm1c^* mice with the *Tnt-Cre* line (Supplemental Figure 2H). Reverse transcription PCR (RT-PCR) confirmed heart-specific deletion of exons 7–10 (Supplemental Figure 2N) resulting in a premature stop codon (Supplemental Figure 1, K–M, and Supplemental Figure 2H). The *Slc5a6^cKO^* mice were observed at the expected numbers at weaning (Supplemental Figure 2O).

Histological analysis at E15.5 revealed that *Slc5a6^cKO^* embryos displayed no developmental cardiac abnormalities (Figure 2, A and B). *Slc5a6^cKO^* mice were phenotypically normal at birth and into early adulthood, but by 20 weeks there was a significant decrease in body weight (Supplemental Figure 2P). Sudden death occurred in *Slc5a6^cKO^* mice at 26 weeks (*n* = 3), and histological analysis revealed cardiac enlargement, with visibly enlarged atria and ventricles (Figure 2, C–F). To comply with local ethical requirements, subsequent litters were euthanized at or before 20 weeks of age. At 14 weeks, cardiomyocyte hypertrophy was observed in both the right and left ventricles (Figure 2, G–I). At 20 weeks, no differences were observed in heart weight–to–body weight or to tibia length ratios (Figure 2J and Supplemental Figure 2Q). Fibrosis was significantly increased 3-fold throughout the ventricular walls of the *Slc5a6^cKO^* hearts compared with control hearts (Figure 2, K–M). An increase in atrial natriuretic peptide (*Nppa*) and a decrease in α-cardiac myosin heavy chain (*Myh6*) expression were observed in *Slc5a6^cKO^* hearts at 20 weeks (Supplemental Figure 2, R and S).

Thus, *Slc5a6^cKO^* mice exhibit the key features of cardiomyopathy, with a cardiac enlargement at 26 weeks, preceded by cardiomyocyte hypertrophy, fibrosis, and activation of fetal gene expression.

### Cardiac function is severely reduced in Slc5a6^cKO^ mice.

Cardiac magnetic resonance (CMR) imaging and 3-lead electrocardiography (ECG) readings were used to assess the functional consequences of structural abnormalities. CMR analysis at 14 weeks revealed a significant reduction in stroke volume (SV) and the percentage change in left ventricular wall thickness in *Slc5a6^cKO^* mice (Supplemental Figure 3, A–F). At 20 weeks, quantification of left ventricular volumes (Figure 3A) showed significant decreases in EF, SV, and cardiac output (CO), and an increase in end systolic volume (ESV) in *Slc5a6^cKO^* mutants (Figure 3, B–E, and Supplemental Table 1). No change in end diastolic volume (EDV) was observed (Figure 3F). A significant change in left ventricular wall thickness between diastole and systole, confirming normal systolic thickening of the left ventricular wall, was observed in the control hearts, but was absent in the *Slc5a6^cKO^* mutants (Figure 3G). Together, these findings are consistent with an early systolic dysfunction phenotype in *Slc5a6^cKO^* hearts at 20 weeks that precedes overt chamber dilation.

ECG was performed on control and *Slc5a6^cKO^* mice at 8 and 20 weeks to monitor cardiac electrical activity as an indicator of cardiac function in vivo, to track cardiac conduction changes longitudinally. As in human ECG, in mice, atrial depolarization appears as the P wave followed by the PR interval, representing the time taken for the electrical impulse to travel from the atria to the ventricles. Ventricular depolarization forms the QRS complex. In mice, the J wave appears as a distinct deflection immediately following the QRS complex, and transitions into the T wave, marking the progression from early to late ventricular repolarization (23). These parameters are all clear in the ECG traces from 20-week control mice (Figure 4A). In comparison, the PR interval in 20-week *Slc5a6^cKO^* mice was visually extended, and the J wave was missing (arrow in Figure 4B). *Slc5a6^cKO^* mutants showed a significantly reduced heart rate at both ages (Figure 4, E and I). Correspondingly, PR intervals were significantly prolonged, increasing from 0.051 ± 0.001 seconds at 8 weeks to 0.063 ± 0.002 seconds (*P* = 0.0001) at 20 weeks in *Slc5a6^cKO^* mice, compared with controls (Figure 4, F and J, and Supplemental Figure 3G), indicating worsening atrioventricular (AV) conduction.

QRS morphology also deteriorated with age. Although QRS duration was normal at 8 weeks, it became significantly widened by 20 weeks (Figure 4, G and K). In contrast, R wave amplitude was significantly reduced at both ages (Supplemental Figure 3, H and I). J waves were present in only 23.5% of *Slc5a6^cKO^* mutants at 8 weeks and were completely absent by 20 weeks (Figure 4, H and L, Supplemental Figure 3, O and P, and Table 1). In addition, S waves were absent in 28.6% of the *Slc5a6^cKO^* mutants (Supplemental Figure 3Q and Table 1).

AV conduction and arrhythmia burden also deteriorated with age. By 20 weeks, high-degree AV block with missed QRS complexes occurred in 21.4% of *Slc5a6^cKO^* mutants, and ventricular ectopy was observed in 28.6% of *Slc5a6^cKO^* mutants, and neither were observed in any other genotypes (Supplemental Figure 3, R and S). In 3 mice, ventricular ectopic beats constituted 20% or more of all QRS complexes, indicating substantial ventricular electrical instability. Thus, all *Slc5a6^cKO^* mutants had ECG abnormalities at 20 weeks, with about a quarter demonstrating severe and life-threatening arrhythmia.

The cardiac-specific deletion of *Slc5a6*, therefore, caused anatomical, histological, and functional cardiac changes consistent with cardiomyopathy, resulting in progressive cardiac dysfunction and sudden death by 26 weeks.

### Impaired biotin and pantothenic acid transport in Slc5a6^cKO^ mice.

Biotin, a cofactor for carboxylases, and pantothenic acid, required for the synthesis of coenzyme A (CoA), are both transported by SMVT (24, 25). To show that vitamin transport was reduced in *Slc5a6^cKO^* hearts, we assessed these vitamin-dependent metabolic pathways.

Analysis of heart tissue by liquid chromatography coupled with quadrupole time-of-flight mass spectrometry (LC-MS-qTOF) revealed significantly increased levels of 3-HIA and 2-methylcitric acid (2-MCA) in *Slc5a6^cKO^* hearts, indicative of MCC and propionyl-CoA carboxylase (PCC) deficiencies, respectively (Figure 5, A and B).

Flow injection analysis coupled with tandem MS (FIA-MS/MS) of the plasma acylcarnitine profile further supported disrupted carboxylase function. Free carnitine (C0), 3-hydroxyisovaleryl-carnitine (C5OH), and the C5OH/C0 ratio were all significantly increased, which are hallmarks of MCC and biotinidase deficiency (Figure 5, C–E). Additional increases in C3DC, C6, C8, and C14 (Figure 5, F–I) reflect accumulation of mitochondrial acyl-CoA intermediates caused by impaired β-oxidation and leucine catabolism, forcing the excess acyl groups to be diverted into the carnitine pool and exported as acylcarnitines.

Additionally, cardiac levels of pantothenic acid and its derivatives phosphopantothenic acid and phosphopantetheine were significantly decreased, leading to impaired CoA synthesis, as evidenced by reduced CoA-glutathione (Figure 5, J–M, and Supplemental Table 2).

Western blot analysis confirmed a significant reduction in MCC and PCC biotinylation in *Slc5a6^cKO^* hearts at 5 weeks (before any structural or functional cardiac differences were observed) and 20 weeks (experimental endpoint) (Figure 5, N and P, and Supplemental Figure 4, A and C), which was unchanged in the liver (Figure 5, O and Q, and Supplemental Figure 4, B and D). Pyruvate carboxylase (PC) levels were unaffected (Figure 5, S–W, and Supplemental Figure 4, A–D). Consistent with findings in patients, the *Slc5a6^cKO^* mice showed elevated C5OH and 3-HIA, hence reproducing the hallmark metabolic changes seen in *SLC5A6* deficiency (10, 15). Together, these results indicate a heart-specific reduction in biotin and pantothenic acid transport in *Slc5a6^cKO^* mice.

### Vitamin supplementation ameliorates the DCM phenotype.

As Patient II-5 in Family 2 responded well to early vitamin treatment, we tested whether vitamin supplementation improved cardiac function in *Slc5a6^cKO^* mice by supplementing drinking water and diet with biotin and pantothenic acid: biotin at approximately 120 mg/kg and pantothenic acid at approximately 2,089 mg/kg, compared with 0.4 mg/kg and 33 mg/kg available in the normal standard diet. This represents an approximately 300- and approximately 63-fold increase, respectively. These doses are equivalent to those used in humans using body surface area scaling and remain substantially below the LC_50_ thresholds for both vitamins (Supplemental Table 3) (26). Supplementation was initiated in parental mice before conception, continued throughout gestation and provided long-term to the offspring. Maintenance of normal levels of MCC and PCC biotinylation in 20-week hearts of supplemented *Slc5a6^cKO^* mice (Figure 5R and Supplemental Figure 4E) indicated successful vitamin uptake.

Vitamin-supplemented *Slc5a6^cKO^* mice survived to 40 weeks, at which time the mice were electively culled. Vitamin supplementation did not affect body weight at 20 or 40 weeks (Supplemental Figure 5, A–F), no excessive fibrosis in *Slc5a6^cKO^* hearts was seen (Figure 2, N–P, and Supplemental Figure 5, G–K), and the expression of *Nppa* and *Myh6* remained normal at 20 weeks (Supplemental Figure 2, R and S). There were no discernible differences in cardiac function (EF, SV, CO, ESV, EDV) or percentage change in left ventricular wall thickness, between vitamin-supplemented control and vitamin-supplemented *Slc5a6^cKO^* mice (Figure 3, B–G), and all ECG parameters were no longer significantly different between control and *Slc5a6^cKO^* mice at 8 and 20 weeks (Figure 4, D–L, and Supplemental Figure 3, H and I). No AV block or ventricular ectopy was observed in vitamin-supplemented *Slc5a6^cKO^* mice (Table 1). At 40 weeks, all the cardiac electrical measurements were similar between control mice on a normal diet and vitamin-supplemented control and *Slc5a6^cKO^* mice (Supplemental Figure 3, J–N). Vitamin supplementation, therefore, prevented the cardiomyopathy phenotype observed in *Slc5a6^cKO^* mice and had no long-term impact on cardiac function.

### Three-dimensional mitochondrial morphology is increased in Slc5a6^cKO^ hearts.

As changes in mitochondrial morphology are associated with metabolic dysfunction and disease progression (27, 28), we analyzed the ultrastructure of cardiac mitochondria using TEM. As conventional 2-dimensional descriptors do not fully capture changes in mitochondrial morphology, we quantified morphology using the mitochondrial complexity index (MCI) (29), a 3-dimensional (3D) metric that integrates measures of mitochondrial volume, surface irregularity, and branching patterns. The MCI increases with greater branching, elongation, and irregularity of mitochondrial shape.

At 20 weeks, control hearts presented with predominantly spherically shaped mitochondria, with minimal branching or complex shapes, and formed parallel clusters along longitudinal myofibers (Figure 6, A and C, and Supplemental Figure 6, A–D). In contrast, mitochondria in *Slc5a6^cKO^* hearts were irregularly shaped and arranged randomly between myofibrils, with evidence of mitochondrial degradation (Figure 6, B and D, and Supplemental Figure 6, E–L). The MCI was significantly increased in *Slc5a6^cKO^* hearts, with a corresponding significant decrease in volume (Figure 6, I and J, and Supplemental Table 4). The *Slc5a6^cKO^* hearts, therefore, had smaller mitochondria with more complex shapes, including excessive elongation, branching, and nanotunnels (Supplemental Figure 6, M–P). This is further represented by the significant difference in the lines of best fit for controls versus *Slc5a6^cKO^* hearts on the bivariate plot (*P* < 0.0001; Figure 6K) and a shift to the right in the cumulative frequency distributions (Figure 6L).

To establish whether the changes in mitochondrial morphology were associated with early indicators of disease, the mitochondria were also investigated at 8 weeks. The overall arrangement of the parallel clusters of the mitochondria along the myofibrils was comparable between control and *Slc5a6^cKO^* mice (Supplemental Figure 7, A and B). There was, however, a significant increase in the MCI of mitochondria in *Slc5a6^cKO^* hearts compared with controls, indicating the mitochondria had a more complex shape, although there was no change in volume (Supplemental Figure 7, C, D, and I–L, and Supplemental Table 4). No significant change in mitochondrial DNA copy number was observed (Supplemental Figure 7, Q and R).

Cardiac mitochondrial morphology was then assessed in vitamin-supplemented mice. At 20 weeks, the overall organization and alignment of mitochondria was similar between vitamin-supplemented control and *Slc5a6^cKO^* hearts. There was a significant reduction in MCI of mitochondria in *Slc5a6^cKO^* hearts compared with controls, indicating that mitochondria were uniform in size and less complex, and there was no change in volume (Figure 6, E–H, M, and N, and Supplemental Table 4), suggesting that vitamins prevented the abnormal mitochondrial morphology. The bivariate plot of mitochondrial MCI versus volume and cumulative MCI frequency showed no differences between vitamin-supplemented control and *Slc5a6^cKO^* hearts at 20 weeks (Figure 6, O and P). At 8 weeks, there was a small yet significant decrease in MCI but not volume (Supplemental Figure 7, E–H, M, and N, and Supplemental Table 4). Interestingly, in vitamin-supplemented mice at 8 weeks, the correlation between the spread of the mitochondria was similar between control and *Slc5a6^cKO^* hearts, but still significantly different (*P* = 0.0084; Supplemental Figure 7, O and P).

Thus, the subtle change in mitochondrial complexity in *Slc5a6^cKO^* hearts at 8 weeks coincides with the early indication of cardiac dysfunction in *Slc5a6^cKO^* mice. As DCM progresses towards functional decline at 20 weeks, the mitochondria become more complex in shape and is associated with a corresponding increase in mitochondria degradation. Vitamin supplementation mitigates this effect in *Slc5a6^cKO^* mice.

### Early disruption of the cardiac proteome precedes cardiomyopathy in Slc5a6^cKO^ mice.

To elucidate the cellular mechanism driving cardiomyopathy progression in the *Slc5a6^cKO^* mice, we performed an unbiased proteomics analysis to identify altered signaling pathways at 8 weeks before the overt cardiomyopathy phenotype was evident. Protein was extracted from hearts of control and *Slc5a6^cKO^* mice maintained on a normal diet and a vitamin-supplemented diet (*n* = 5 per group).

Analysis of differential protein expression, using principal component analysis and a hierarchical heatmap plot, showed that the *Slc5a6^cKO^* samples formed a distinct group compared with the control and vitamin-supplemented samples (Figure 7, A and B).

In *Slc5a6^cKO^* hearts on a normal diet, a total of 1,459 proteins showed significant differential expression (599 downregulated, 860 upregulated, *P* < 0.05) compared with controls (Figure 7C). For the vitamin-supplemented mice, only 153 proteins showed significant differential expression (81 downregulated, 72 upregulated, *P* < 0.05) in *Slc5a6^cKO^* hearts compared with controls (Figure 7D). Of the proteins differentially expressed under a normal diet, only 124 of those proteins remained significantly differentially expressed following vitamin supplementation (Figure 7E). There was no significant difference in protein expression between control mice on the normal diet or the vitamin-supplemented diet.

Pathway analysis was performed on the significantly up- and downregulated proteins combined. From *Slc5a6^cKO^* mice on a normal diet, in the top 15 significantly enriched pathways (Figure 7F), the upregulated pathways included the “sirtuin signaling pathway” and “collagen biosynthesis and modifying enzymes,” which are both linked to increasing fibrosis (30, 31), and “granzyme A signaling,” which correlates with the upregulation of mitochondrial dysfunction pathways (32). Downregulated pathways were predominantly related to energy metabolism: “respiratory electron transport,” “mitochondrial translation,” “oxidation phosphorylation,” “complex I biogenesis,” “branched-chain amino acid [BCAA] catabolism” (specifically valine and isoleucine degradation), and “fatty acid β-oxidation.” The hematoma resolution signaling pathway is linked to genes involved in peroxisomal β-oxidation (33). In contrast, vitamin supplementation dramatically reduced the number of enriched pathways in *Slc5a6^cKO^* hearts (Figure 7G), with downregulation only identified in peroxisome-related pathways. There was no upregulation of other known vitamin-related transporters, including monocarboxylate transporter 1 (MCT1), which transports biotin in specific cell types such as immune cells and skin, in *Slc5a6^cKO^* hearts on a normal or vitamin-supplemented diet.

Energy metabolism involves several interconnected pathways. Analysis of different pathways showed that the majority of proteins linked to the TCA cycle, fatty acid β-oxidation, BCAA catabolism, and the respiratory electron transport chain (ETC) were all downregulated in the *Slc5a6^cKO^* hearts on a normal diet, with a corresponding increase in proteins involved in glycolysis (Figure 8A), which is a common compensatory response in heart failure (34).

Western blot analysis of heart tissue confirmed the downregulation of 2 keys enzymes required in β-oxidation, medium-chain acyl-CoA dehydrogenase (MCAD) and hydroxyacyl-CoA dehydrogenase trifunctional multienzyme complex subunit α (HADHA) (Figure 8, B and C, and Supplemental Figure 8). Each mitochondrial complex in the ETC, complex I to V, is made from multiple subunits. Notably, from the subunits detected in the proteomics experiment, the components exhibited widespread reduced expression in *Slc5a6^cKO^* mutants compared with controls, with the following percentage of subunits showing decreased expression: complex I 90.2%, complex II 100%, complex III 77.8%, complex IV 77%, and complex V 76.5% (*q* < 0.05, all fold changes; Supplemental Table 5). Remarkably, vitamin supplementation substantially prevented these deficits, reducing the proportion of downregulated subunits to only 2 in complex I (NDUFA11 and NDUFS6) and 1 in complex IV (MT-CO3) in vitamin-supplemented *Slc5a6^cKO^* hearts compared with controls. Quantitative real-time PCR (qPCR) analysis of representative genes from the subunits of each complex (Supplemental Table 5) confirmed significant downregulation of subunits in complexes I, II, IV, and V in *Slc5a6^cKO^* hearts (Figure 8, D–H), with no significant differences detected in vitamin-treated hearts (Figure 8, I–M).

Integrated analysis of proteomic and metabolomic datasets from 8-week-old control and *Slc5a6^cKO^* hearts revealed coordinated changes across multiple energy-metabolism pathways, including glycolysis, pyruvate oxidation, β-oxidation, TCA cycle, and BCAA catabolism (Supplemental Figure 9 and Figure 9). All 5 components of the pyruvate dehydrogenase (PDH) complex were downregulated, potentially reducing the capacity for pyruvate oxidation via this lipoic acid– and CoA-dependent pathway. In parallel, reduced abundance of key fatty acid transport and β-oxidation enzymes, together with accumulation of medium- and long-chain acylcarnitines, is consistent with incomplete mitochondrial fatty acid oxidation and diminished contribution to acetyl-CoA production. The TCA cycle also exhibited coordinated downregulation of multiple enzymatic components, suggesting reduced cycle capacity to oxidize acetyl-CoA. Finally, BCAA catabolism was broadly suppressed, with reduced expression of enzymes involved in leucine, isoleucine, and valine oxidation and evidence of propionyl-CoA overflow, including accumulation of 2-methylcitrate.

## Discussion

In this study, we have identified impaired *SLC5A6* function as a genetic cause of DCM in 2 unrelated families. Clinical outcomes in these families highlight the critical importance of early intervention; timely and sustained vitamin supplementation prevented the progression to end-stage heart failure and the need for heart transplantation, whereas delayed treatment was associated with irreversible cardiac decline requiring transplantation. Using a cardiac *Slc5a6–*deleted mouse model, *Slc5a6^cKO^*, we confirmed *Slc5a6* as a gene associated with cardiomyopathy, with clinical features of DCM in patients, linked to abnormal mitochondria and impaired energy metabolism that are prevented with vitamin supplementation.

In *Slc5a6^cKO^* mice, inactivation of *Slc5a6* specifically in cardiomyocytes resulted in a lethal phenotype with characteristic anatomical, functional, and histological features of cardiomyopathy, including enlarged hearts at the time of death (~26 weeks) (35–37). *Slc5a6^cKO^* mice were phenotypically normal up to 8 weeks, from which time point abnormal ECG readings were identified, demonstrating early indicators of DCM (38, 39). *Slc5a6^cKO^* mice had a lower heart rate, consistent with the known inverse correlation between heart rate and PR interval (40). The CMR imaging data at 14 and 20 weeks (~6 weeks prior to death) revealed systolic dysfunction in *Slc5a6^cKO^* hearts from 20 weeks, evidenced by reduced SV, CO, and EF, alongside increased ESV but unchanged EDV. We also observed early myocardial fibrosis and reactivation of the fetal gene program (41, 42) prior to detectable changes on CMR, further supporting the concept that electrical and histological remodeling precede overt structural dilation (43, 44). Notably, the absence of EDV change does not preclude ventricular dilation; rather, it may reflect a transitional stage preceding overt structural remodeling of the left ventricle. Therefore, as the analysis of the *Slc5a6^cKO^* mice was restricted to an end point of 20 weeks, this model reflects the transitional and progressive nature of cardiomyopathy with features consistent with DCM. Functional evidence of left ventricular dilation was not assessed but may become apparent after 20 weeks in *Slc5a6^cKO^* hearts as further adverse remodeling progresses. Furthermore, ECG findings indicated the presence of arrhythmias and conduction defects that can be associated with mortality (45, 46); however, without lung weights from the 20-week cohort, congestive heart failure cannot be excluded.

Increased fibrosis causes myocardial stiffness and is a characteristic feature of DCM (47, 48). In human DCM, there is an initial adaptive response whereby an increase in collagen fibers boosts growth of the myocardium to enhance contractile force (49), and this correlates with our proteomic data, showing an increase in collagen-related pathways in mutant mice at 8 weeks. However, over time, this process becomes pathological, resulting in impaired cardiomyocyte contractility, causing stiffening of the myocardium, reducing SV and EF until heart failure occurs.

Metabolomic analysis of *Slc5a6^cKO^* mouse hearts confirmed deficiencies in both biotin and pantothenic acid metabolism consistent with the loss of *SLC5A6*-mediated vitamin transport. *SLC5A6* facilitates the uptake of dietary biotin and pantothenic acid across the cell membrane. Biotin is an essential cofactor for several carboxylases: acetyl-carboxylase (ACC), PCC, MCC and PC. Pantothenic acid is required for the synthesis of CoA (24, 25).

Due to biotin deficiency, MCC activity, which is required for leucine metabolism, is reduced and the breakdown of 3-methylcrotonyl-CoA occurs via an alternative pathway, leading to the accumulation of C5OH and 3-HIA (22, 50, 51), metabolites that were elevated in *Slc5a6^cKO^* hearts and in the patients. Similarly, PCC catalyses the conversion of propionyl-CoA to methylmalonyl-CoA, which is also a crucial step in the metabolism of BCAA (including isoleucine and valine). PCC deficiency leads to the accumulation of 2-methylcitrate (52), a metabolite that was elevated in the *Slc5a6^cKO^* mouse hearts. Furthermore, 3 downregulated pathways identified by proteomic analysis are linked to BCAA catabolism. Additionally, pantothenic acid is converted through a series of steps into CoA. Pantothenic acid itself and 2 of its downstream derivatives, phosphopantothenic acid and phosphopantetheine, were all decreased in the *Slc5a6^cKO^* mouse hearts.

These findings confirm that cardiomyocyte-specific *Slc5a6* deletion disrupts vitamin uptake and mirrors the metabolic abnormalities observed in patients with *SLC5A6* mutations, as well as those with PCC and MCC mutations. Reduced vitamin transport into the heart, therefore, leads to elevated BCAA levels and reduced expression of BCAA catabolic enzymes that have been observed in DCM patients (53). Enhanced BCAA oxidation in a transverse aortic constriction mouse model improved cardiac function (53), which supports our hypothesis that biotin and pantothenic acid supplementation could serve as an alternative therapeutic strategy to improve cardiac function by preventing a block in BCAA catabolism.

Collectively, these omic-level changes are consistent with impaired mitochondrial energy metabolism in *Slc5a6^cKO^* hearts and point to multiple nodes of metabolic vulnerability. While proteomic and metabolomic analyses do not directly assess metabolic flux or enzyme activity, the concordance between reduced abundance of key metabolic enzymes and accumulation of pathway-specific metabolites supports biologically meaningful disruption of substrate utilization. These integrated datasets therefore provide a strong framework for understanding disease progression and underscore the importance of functional and interventional studies to establish causality.

Of the patients with *SLC5A6* mutations reported in the literature, 64% received different combinations of vitamin supplementation at different stages of disease presentation, resulting in clinical improvements (8–12, 14–20). In Family 1 reported here, for Patient II-1 no vitamin treatment was given, and she died from heart failure before the opportunity of a heart transplant arose. For her sibling, Patient II-2, vitamin treatment was not initiated until 14 years of age, 7 years after the heart transplant. Modest improvement in development and growth was seen over the past 6 years, associated with much improved control of her epilepsy. In Family 2 (15), biotin supplementation was initiated 3 weeks after admission for heart failure, and then pantothenic acid was added at day 64, leading progressively to the reversal of the cardiac phenotype and maintained normal cardiac function.

To evaluate whether vitamin supplementation could affect disease progression in our *Slc5a6^cKO^* mouse model, dietary biotin and pantothenic acid were given to the parents before conception and to the pups after weaning. The vitamins were administered at high but safe pharmacological concentrations comparable to doses given to patients (26). It has been shown that at physiological levels, water-soluble vitamins, such as biotin and pantothenic acid, are taken up by high-affinity transporters, but at elevated concentrations they can also cross the cell membranes by passive diffusion (54, 55), bypassing the need for a functional vitamin transporter in *Slc5a6^cKO^* mice. The daily adequate intake (AI) for biotin from birth to 3 years is 5–8 μg, rising to 30 μg for adults, and the corresponding AI for pantothenic acid is 1.7–2 mg, rising to 5 mg for adults (56). Interestingly, in the clinical setting, Patient II-5 from Family 2 showed no response to an initial 10 mg dose of biotin but experienced rapid clinical improvement following an increase to 15 mg, supporting the notion that diffusion-mediated uptake can be therapeutically relevant at higher concentrations. Lipoic acid was not supplemented in our mouse model, as it is synthesized endogenously in the mitochondrial matrix and does not require dietary intake in mammals, unlike biotin and pantothenic acid. Remarkably, vitamin-supplemented mice were phenotypically normal and showed no evidence of cardiac dysfunction or fibrosis and survived until they were electively culled at the end of the study period (40 weeks). These findings underscore the potential of early high-dose vitamin therapy to mitigate cardiac pathology in the absence of *SLC5A6* function. Future research should explore the effects of initiating treatment at different developmental stages, as this could inform optimal therapeutic windows in humans, including whether cardiac dysfunction can be reversed, as well as whether both biotin and pantothenic acid are required. Importantly, our findings suggest that incorporating *SLC5A6* into prenatal screening could facilitate early diagnosis and timely intervention, potentially improving long-term clinical outcomes.

Previously, there had not been a focused analysis of the heart in patients with *SLC5A6* mutations. The results presented here provide a detailed overview of the impact of vitamin loss specifically within the cardiomyocytes on cardiac function and highlights that the underlying initiation of clinical symptoms may be due to a defect in energy metabolism from mitochondrial abnormalities. Imbalances in energy generation or energy use are thought to be key factors in the pathogenesis of DCM (57), which ultimately leads to heart failure (58). Given the critical role of mitochondria in energy metabolism, changes in mitochondrial dynamics and function have been associated with cardiovascular diseases and heart failure (4). However, it is unknown whether the metabolic energetic dysfunction is a cause or a consequence of disease in DCM. Given the link between the vitamins transported by SMVT and mitochondrial energy metabolism, the morphology of mitochondria was analyzed, as changes in mitochondrial structure can be a pathological factor in disease progression (27) and alterations are known to be associated with functional deficiencies (28). In cardiomyocytes, the mitochondria are arranged in a highly ordered arrangement, forming rows parallel to the myofibrils (59), but this organized assembly was lost in *Slc5a6^cKO^* hearts. The MCI, an indicator of mitochondrial morphology (27), was considerably increased in the *Slc5a6^cKO^* hearts by 20 weeks, with increased branching and abnormal donut-shaped mitochondria that are indicative of cellular stress (60). Mitochondrial morphology could be an early indicator of disease onset, as smaller yet significant changes in MCI were observed at 8 weeks, alongside the downregulation of key energy metabolism pathways, prior to the onset of an overt DCM phenotype. Interestingly, vitamin supplementation prevented the long-term changes in mitochondrial morphology in the *Slc5a6^cKO^* hearts. This was also reflected in proteomic data that showed there was no longer a downregulation in mitochondrial metabolism pathways.

Future work is required to determine whether impaired energy metabolism represents the primary pathogenic consequence in *Slc5a6^cKO^* hearts and to establish whether anaplerotic pathways are also affected, as suggested by deficits consistent with MCC dysfunction. Although our study focuses on mitochondrial abnormalities, biotin and pantothenic acid also support several extramitochondrial pathways, including cytosolic ACC1 activity and CoA-dependent processes in the cytosol, peroxisomes, endoplasmic reticulum, and nucleus. We, therefore, cannot exclude the possibility that altered nonmitochondrial metabolism contributes to the pathological phenotype observed. Elucidating these extramitochondrial effects therefore represents an important priority for future investigation.

Furthermore, as *TnT-Cre* is active from E7.5, coinciding with early cardiac development and the onset of rapid cardiomyocyte expansion, any preexisting Slc5a6 protein would be expected to be diluted through subsequent embryonic cell divisions, although some persistence due to protein stability cannot be fully excluded.

Our findings underscore the critical importance of early diagnosis and intervention in *SLC5A6*-related DCM. Vitamin supplementation, when administered early, not only prevents the onset of mitochondrial abnormalities but also halts the progression of cardiac dysfunction, offering a promising therapeutic approach for patients with metabolism-related cardiac diseases. The *Slc5a6^cKO^* mouse model has proven to be an invaluable tool for unravelling the complex relationship between vitamin transport, mitochondrial energy metabolism, and cardiac dysfunction. These results pave the way for emerging strategies focused on enhancing mitochondrial function, such as vitamin supplementation, which could preserve cardiac function by boosting energy metabolism (61), potentially transforming the treatment landscape for DCM and other energy metabolism–related heart conditions.

## Methods

### Sex as a biological variable

Our study examined both male and female animals and the data were combined, as there were no differences between the sexes.

### Animals

*Slc5a6* transgenic mice (*Slc5a^Tm1a(EUCOMM)Wtsi^*) were obtained from the EUCOMM/IMPC consortium (Supplemental Figure 2H). The construct included a neomycin (neo)/LacZ cassette flanked by FRT and loxP sites, inserted between exon 6 and 7. Additional loxP sites were inserted upstream (5′) of exon 7 and downstream (3′) of exon 10, to enable targeted removal of these 4 exons within *Slc5a6* using *Cre* recombinase. *Slc5a6^+/Tm1a^* mice were mated to generate homozygous *Slc5a6^Tm1a/Tm1a^* mice. In parallel, the neo/LacZ cassette was removed by mating with FLP recombinase mice (62) to generate *Slc5a6^Tm1c^* mice. Homozygous *Slc5a6^Tm1c/Tm1c^* were further mated with *TnT-Cre* mice (63) to delete the function of *Slc5a6* specifically within cardiomyocytes from E7.5, to produce *Slc5a6* cardiac conditional knockout *Slc5a6^Tm1c/Tm1c^;TnT-Cre^+/–^* (referred to as *Slc5a6^cKO^*) mice (Supplemental Figure 2H). All mice were maintained on a C57BL/6J background.

The normal mouse diet contained 0.4 mg/kg biotin and 33 mg/kg pantothenic acid. For vitamin supplementation, breeding cages were provided with vitamin-supplemented mouse diet (TD.200233 custom diet, ENVIGO) containing 120 mg/kg biotin and 2089 mg/kg pantothenic acid, in food pellets. Additionally, 1 mM biotin and 1 mM pantothenic acid were supplied through drinking water. Vitamin supplementation was initiated prior to conception and maintained throughout the offsprings’ life (Supplemental Table 3).

### RT-PCR and qPCR

Total RNA was isolated from mouse tissue using the ReliaPrep RNA Miniprep kit (Promega). cDNA was synthesized using the high-capacity cDNA reverse transcription kit (Thermo Fisher Scientific). For validation of the *Slc5a6^cKO^* mouse model, RT-PCR was used to confirm the removal of exons 7–10 specifically in the heart only.

qPCR was performed using SYBR Green Master Mix (Thermo Fisher Scientific) in triplicate on a QuantStudio 7 Real-Time PCR System. Primers are listed in Supplemental Methods. Data were analyzed using the comparative Ct method (64) and were normalized to *Gapdh* and *Actb* housekeeping genes, and data are shown as the fold change relative to control samples.

### Histology

Hearts were collected following cervical dislocation and cryoprotected in 7.5%–15% sucrose solution for 2 hours before freezing in OCT or fixed in 4% paraformaldehyde and embedded in paraffin wax. Serial tissue sections (10 μm) were cut and stained with H&E and Picrosirius red using standard histological protocols and imaged on a Zeiss Axio Imager using bright-field mode. Details of the quantification of fibrosis are in the Supplemental Methods.

### Protein expression

#### Immunofluorescence.

The human embryonic and fetal material was provided by the Joint MRC/Wellcome Trust Human Developmental Biology Resource (https://www.hdbr.org/). Human embryonic sections were stained by immunofluorescence using a primary antibody against SLC5A6 (26407-1-AP, Proteintech; 1:100 dilution) and the nuclei were counterstained with DAPI. Images were acquired on a Zeiss Axio Imager.

Mouse heart sections were stained with wheat germ agglutinin (WGA) conjugated to Alexa Fluor 594 to stain the cell membranes. The cell area of cardiomyocytes was measured using ImageJ analysis software (NIH). Per heart section, 4 region-of-interest images were taken per ventricle, with 3 technical repeats.

#### Western blotting.

Protein expression was quantified by Western blot analysis using standard techniques. Briefly, protein was extracted using protein homogenization buffer (T-PER, Thermo Fisher Scientific, protease inhibitors and phosphatase inhibitors). Samples were homogenized with an electric homogenizer for 30 seconds and centrifuged. Protein lysates (15,000 ng) were denatured in NuPAGE sample buffer and were separated in a 4%–15% Tris-glycine precast gel with 1× Tris-glycine running buffer. Protein was transferred to a PVDF membrane. Biotinylation levels of PCC, MCC, and PC were measured using an HRP conjugate (65), IRDye 800CW streptavidin (Licor), anti-MCAD (Abcam, ab92461; 1:10,000), anti-HADHA (Proteintech, 10758-1-AP, 1:10,000), and anti-GAPDH (Abcam, ab22333; 1:1,000). Membranes were imaged and quantified using fluorescent detection with secondary IRDye-conjugated anti-streptavidin antibodies (IRdye anti-strep 800 for biotinylation and IRdye goat anti-rabbit 680 to detect MCAD, HADHA, or GAPDH) on the LI-COR Odyssey FC.

### Protein modeling

3D protein structures of SLC5A6 were predicted using AlphaFold2 (66) accessed via ColabFold (67). The SLC5A6 amino acid sequence was obtained from the UniProt database (68) (UniProt ID: Q9Y289) and used as the base query sequence for all structure predictions.

To determine the impact of P437L or R253W point mutations on the SLC5A6 structure, residue substitutions were inserted into the SLC5A6 query sequence as well as into the multiple sequence alignment generated by the WT SLC5A6 AlphaFold2 structure prediction (69). The resulting structure with the highest rank was visualized using PyMOL (The PyMOL Molecular Graphics System, version 2.5.5 Schrödinger, LLC. Available from: http://www.pymol.org/pymol).

### Cardiac function: ECG

ECG was performed on anesthetized mice at 8, 10, 14, 20, and 40 weeks using a 3-lead ECG system (PowerLab) (70). ECG was recorded for 3 minutes using the PowerLab data acquisition system (ML866, ADInstruments) and animal bio amp (FE136, ADInstruments) in channel 1. Analysis was performed on parameters including heart rate (BPM), PR interval (seconds), QRS interval (seconds), and J wave amplitude (mV) using LabChart software (AD Instruments). An average of the traces in the third minute was produced in LabChart blinded to genotype.

### Cardiac function: CMR imaging

CMR imaging was performed on anesthetized mice using a 7.0-T horizontal bore Varian microimaging system with a 12 cm microimaging gradient insert (Varian Inc.). Short-axis images were acquired and used to determine cardiac left ventricular function by measuring the left ventricular chamber volume in end systole and end diastole to evaluate stroke volume (mL), cardiac output (mL), and ejection fraction (%) using ImageJ software.

### TEM

Pieces of adult heart (2 × 2 × 2 mm) were collected and fixed in 2% glutaraldehyde and were stained with heavy metal and embedded in 100% resin (71). Heavy metal–stained sections (70 nm) were imaged with a CM100 TEM (FEI) in a longitudinal orientation to assess mitochondrial morphology and sarcomeric structure.

### Serial block face scanning electron microscopy and 3D reconstructions

Samples were stained with heavy metal and embedded in 100% resin. Resin blocks were sectioned using in situ Zeiss Sigma scanning electron microscopy and 250 stacked images were acquired per region of interest (between 2 and 4). Images taken from each region of interest were combined into a stack using ImageJ (2000 × 2000 × 250 pixels) and the contrast was adjusted to visualize mitochondria throughout. Further analysis was performed using Microscopy Image Browser (MIB, University of Helsinki). Each image stack was aligned and individual mitochondria within a cluster along a myofibril was segmented. Segmented mitochondrial clusters were imported into Amira software (Thermo Fisher Scientific) to generate 3D reconstructions. Amira calculated the volume and surface area of each individual mitochondria. These calculations were used to determine the MCI (29). MCI is used as a quantitative measure of morphological complexity and is the 3D equivalent of form factor (branching) and is analogous to sphericity. The MCI is a parameter used to quantify the 3D shape of mitochondria, irrespective of volume. Thus, spherical mitochondria, whether they be small or large, will have a similar MCI value of approximately 1. In contrast, mitochondria that are elongated, branched, fused, or fragmented will have varying MCI values from 0 to infinity.

### Metabolomics

Acylcarnitine analysis of mouse plasma was performed by FIA-MS/MS using isotope dilution after a butanol derivatization step, as part of the routine workflow in the Clinical Biochemical Genetics Laboratory in Brussels. Metabolomic analysis of homogenized frozen heart powder was performed on the upper aqueous fraction after liquid-liquid Folch extraction (72). CoA-glutathione measurement ([M-H] ^–^1071.1748) was performed using an ion-pairing LC-MS-qTOF Agilent 6550 ion funnel analysis (72). Other metabolites (3-HIA [M-H] ^–^117.0562, 2MCA [M-H] ^–^205.0362, pantothenic acid [M-H] ^–^218.1051, PPA [M-H] ^–^298.072, and phosphopantetheine [M-H] ^–^357.088) were measured by Waters Synapt-XS LC-MS-qTOF with an electrospray ionization source in negative mode in a reverse-phase chromatography method using an Acquity Premier HSS T3 2.1 × 100 mm, 1.8 μm column (73).

### Proteomics

Global proteomic profiling was performed using data-independent acquisition (DIA) MS. Proteins were enzymatically digested using a Suspension Trap–based workflow and analyzed by high-resolution LC–MS/MS on an Orbitrap Exploris 480 platform. DIA data were processed using DIANN with a mouse reference proteome, followed by downstream statistical analysis in Perseus. Differential protein abundance between *Slc5a6^cKO^* mutants and controls was assessed within dietary groups, and significantly altered proteins were subjected to pathway analysis using Ingenuity Pathway Analysis (IPA). Detailed experimental procedures are described in the Supplemental Methods.

### Statistics

To determine the distribution of all data, a normality test (Shapiro-Wilk) was performed. For data that were not normally distributed, a non-parametric statistical test was applied (Mann-Whitney or Kruskal-Wallis test with Dunn’s correction for multiple comparisons). Normally distributed data were subject to parametric testing using an unpaired 2-tailed *t* test or 1-way ANOVA with Bonferroni’s correction for multiple comparisons. Mendelian inheritance patterns were assessed with a χ^2^ test. For the mitochondria reconstructions, the slopes of the 2 regression lines (MCI versus volume) were compared in Prism using an Analysis of Covariance (ANCOVA). Differences between the percentages of mitochondria being simple or complex was tested using a 2 × 2 contingency table and Fisher’s exact test. All data are presented as mean ± SEM. A *P* value of less than 0.05 was considered statistically significant. Analyses were performed using GraphPad Prism software (version 10.4.1).

### Study approval

All studies involving animals were performed in accordance with the UK Home Office Animals (Scientific Procedures) Act 1986 and all experiments were approved by Newcastle University Animal Welfare and Ethical Review Body.

### Data availability

The proteomic data are available in the MassIVE Repository at Welcome to MassIVE (ftp://massive-ftp.ucsd.edu/v09/MSV000097747/). A summary of the proteomic data is provided in Supplemental Dataset 1. All data values are provided in the Supporting Data Values file.

## Author contributions

MOF, LCP, RC, ADHM, VH, DR, and JPD conducted experiments and acquired data. MOF, LCP, LS, RER, STK, NM, JPD, and HMP analyzed data. RER, GM, STK, GDR, and JB provided essential resources. HMP, SDB, MOF, LCP, and JPD designed the experiments. HMP and SDB wrote the manuscript. MOF, LCP, RER, GDR, JB, SDB, and HMP edited the manuscript. All authors read and approved the final version of the manuscript.

## Conflict of interest

The authors have declared that no conflict of interest exists.

## Funding support

- British Heart Foundation grant numbers PG/16/105/32659 and PG/24/11744 (awarded to HMP and SDB).
- PhD studentship from Federated Foundation (Borwick Charitable Trust, Charity Number 291528), awarded to HMP and SDB.
- Biotechnology and Biological Sciences Research Council grant numbers BB/M012093/1 and BB/R013942/1 (awarded to the Newcastle University Electron Microscopy Unit).

## Supplementary Material

Supplemental data

Supplemental data set 1

Unedited blot and gel images

Supporting data values

## Figures and Tables

**Figure 1:**
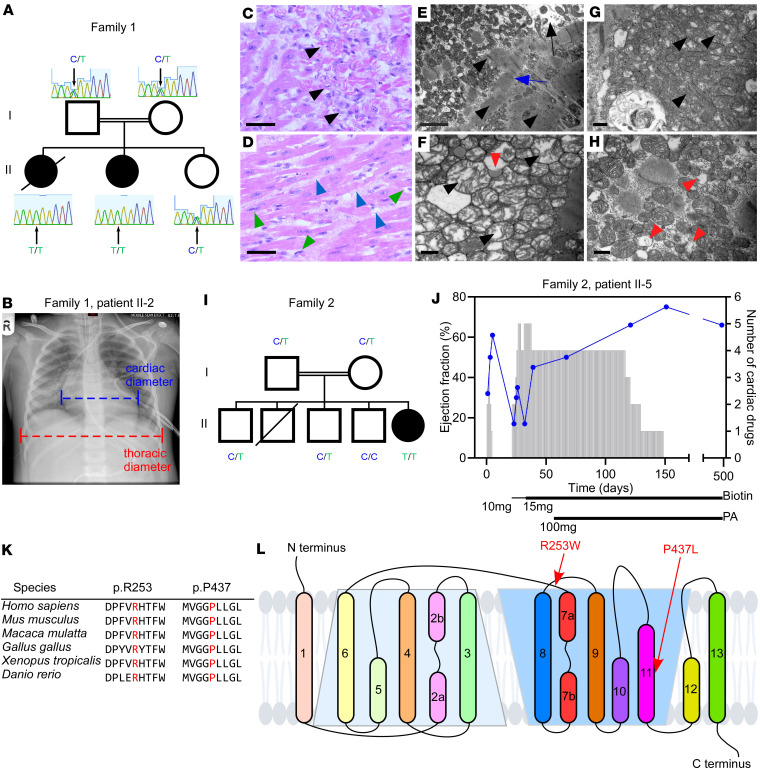
Clinical presentation and identification of the *SLC5A6^R253W^* mutation. (**A**) Pedigree of Family 1. The unaffected consanguineous parents and child II-3 were heterozygous for the *SLC5A6* mutation (C/T) in R253, whereas children II-1 and II-2 both had DCM and were homozygous (T/T). (**B**) X-ray of child II-2 from Family 1, with a cardiothoracic ratio of 0.57, indicating an enlarged heart. (**C**–**H**) Imaging of a left ventricular heart biopsy from child II-2. (**C** and **D**) H&E-stained sections showed myocardial disarray with hypertrophied cardiomyocytes (black arrowheads), hyperchromatic nuclei (green arrowheads), and myofibrillar loss (blue arrowheads). (**E**–**H**) Sarcomeric wasting (black arrow in **E**), sarcomeric disarray (blue arrow in **E**), Z band disarray (black arrowheads in **E**), fragmented mitochondria (black arrowheads in **F** and **G**), and mitochondrial degradation (red arrowheads in **F** and **H**). (**I**) Pedigree of Family 2. The unaffected consanguineous parents were heterozygous for the *SLC5A6* mutation (C/T) in P437, whereas Patient II-5 had DCM and was homozygous (T/T). Child II-2 died at 8 months from heart failure, but no genetic testing had been performed. (**J**) Timeline and treatment of Patient II-5 from Family 2. The ejection fraction (EF, blue line) and the administration of up to 5 cardiac drugs (gray histogram) at different time points are shown. The first day of admission to hospital is classified as day 0. The cardiac drugs were stopped on day 148 when the EF became stabilized. Vitamin treatment with biotin and pantothenic acid (PA) is ongoing, and the EF continues to be stabilized. (**K**) Amino acid residues R253 and P437 are both highly conserved across species. (**L**) Schematic of SMVT. The patient mutation, p.R253W (Family 1), is in the hinge region of the transporter and the p.P437L mutation (Family 2) is located within transmembrane domain 11. Scale bars: 20 μm (**C** and **D**), 2 μm (**E**), and 500 nm (**F**–**H**).

**Figure 2:**
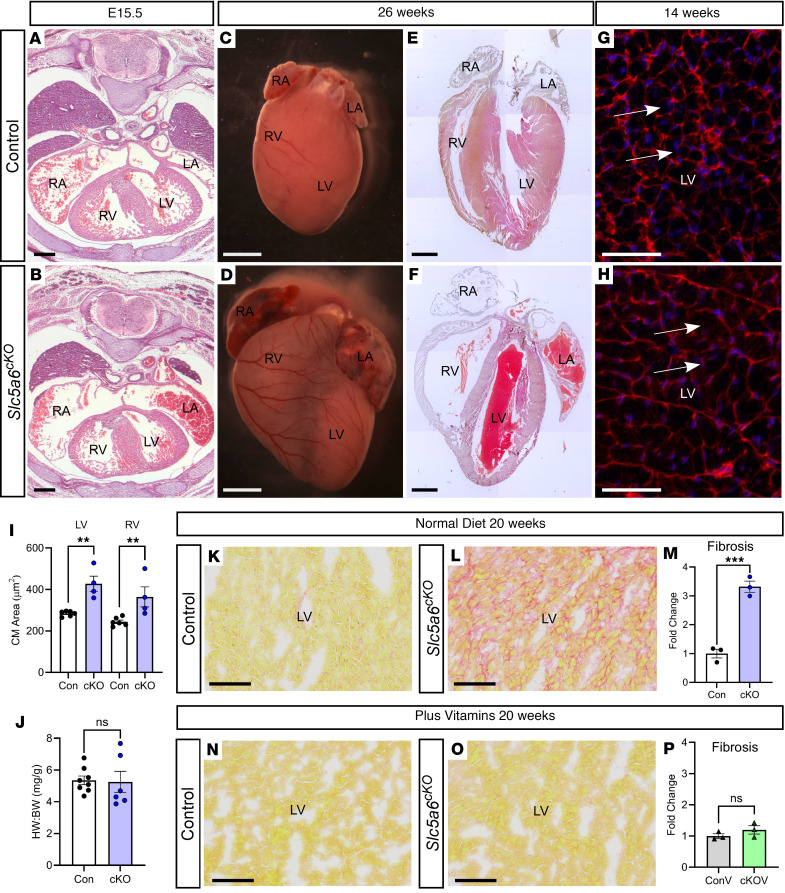
Cardiac-specific *Slc5a6*-KO (*Slc5a6^cKO^*) mouse recapitulates a cardiomyopathy phenotype. (**A** and **B**) Hearts from E15.5 control embryos (**A**) were comparable to *Slc5a6^cKO^* mutant embryos (**B**). (**C**–**F**) *Slc5a6^cKO^* mutant hearts collected at 26 weeks were visibly enlarged (**D** and **F**) compared with controls (**C** and **E**). (**G** and **H**) WGA staining was performed on coronal heart sections of 14-week control (**G**) and *Slc5a6^cKO^* mutant hearts (**H**). (**I**) Cardiomyocytes (CM) were significantly larger in both ventricles in the *Slc5a6^cKO^* mutant (*n* = 4) compared with the control (*n* = 6) hearts. (**J**) No difference in heart weight (HW) to body weight (BW) ratio was observed between control (*n* = 8) and *Slc5a6^cKO^* mutant (*n* = 6) mice at 20 weeks. (**K**–**M**) Fibrosis was increased in *Slc5a6^cKO^* mutant hearts (*n* = 3; **L** and **M**) compared with control hearts (*n* = 3; **K** and **M**) at 20 weeks. (**N**–**P**) Following vitamin supplementation, no increase in fibrosis in *Slc5a6^cKO^* mutants (*n* = 3) compared to control (*n* = 3) mice was observed. Data are represented as mean ± SEM. ns, nonsignificant. **P < 0.01, ***P < 0.001 by 1-way ANOVA with Bonferroni’s correction for multiple comparisons (**I**) or unpaired t-test for two sample comparisons (**M**, **J**, and **P**). RV, right ventricle; LV, left ventricle; RA, right atria; LA, left atria; Con, control; cKO, *Slc5a6^cKO^*; ConV, vitamin-supplemented control; cKOV, vitamin-supplemented *Slc5a6^cKO^*. Scale bars: 500 μm (**A** and **B**), 2 mm (**C** and **D**), 1 mm (**E** and **F**), 50 μm (**G** and **H**), and 100 μm (**K**, **L**, **N**, and **O**).

**Figure 3:**
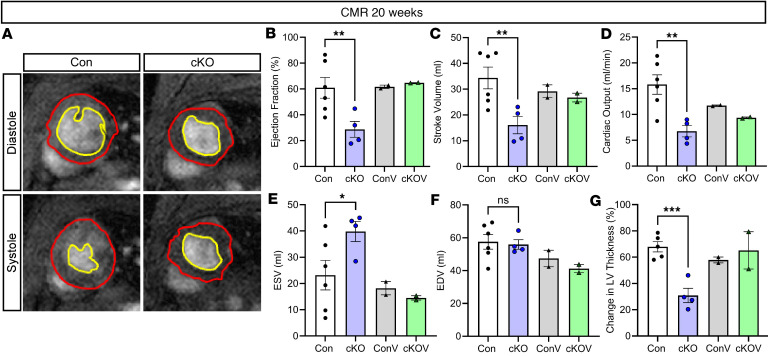
Impaired cardiac contractility and ventricular remodeling in *Slc5a6^cKO^* mice are prevented by vitamin supplementation. (**A**–**G**) Cardiac magnetic resonance (CMR) imaging at 20 weeks. (**A**) Mouse cardiac CMR images of short-axis slice through the mid-heart showing left ventricular diastole and systole, with the epicardium outlined in red and endocardium in yellow. Significant decreases in ejection fraction (**B**), stroke volume (**C**), cardiac output (**D**), end systolic volume (ESV) (**E**), and percentage change in left ventricular (LV) wall thickness at diastole and systole (**G**) were detected in *Slc5a6^cKO^* mutants (*n* = 4) compared with controls (*n* = 6). The end diastolic volume (EDV) showed no difference (**F**). After vitamin supplementation, no apparent differences in cardiac function were seen (*n* = 2 for each genotype). Con, control; cKO, *Slc5a6^cKO^*; ConV, vitamin-supplemented control; cKOV, vitamin-supplemented *Slc5a6^cKO^*. Data are represented as mean ± SEM. ns, nonsignificant. *P < 0.05, **P < 0.01, ***P < 0.001 by 1-way ANOVA with Bonferroni’s correction for multiple comparisons (**B**–**G**) or nonparametric Kruskal-Wallis test with Dunn’s correction for multiple comparisons.

**Figure 4:**
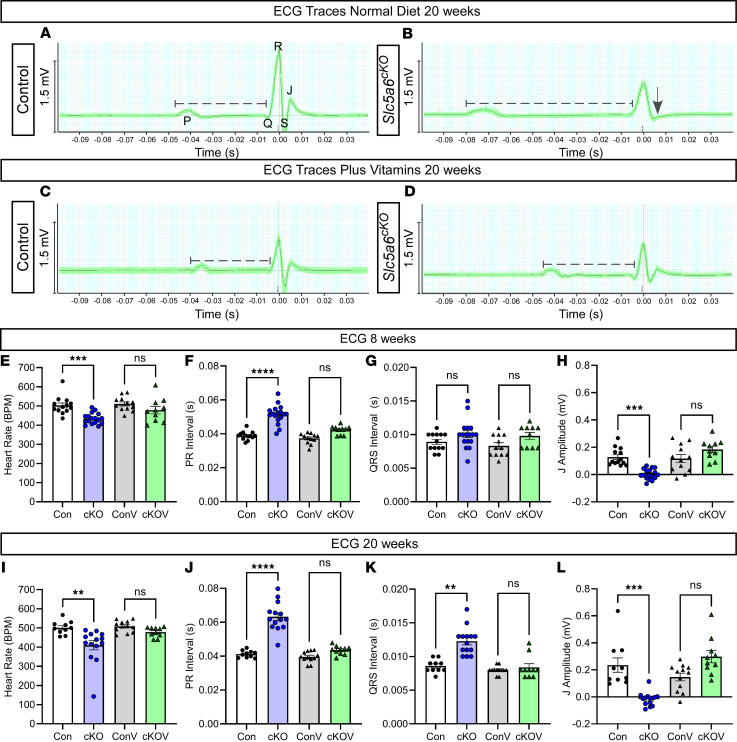
*Slc5a6^cKO^* mice develop progressive electrical conduction abnormalities that are prevented by vitamin supplementation. (**A**–**D**) Representative average ECG traces from 20-week mice. Control (**A**) and vitamin-supplemented control (**C**) mice have a well-defined P wave, QRS complex, and J wave, whereas *Slc5a6^cKO^* mice (**B**) presented with a longer PR interval (dotted line) and loss of the J wave (arrow). ECG traces from vitamin-supplemented *Slc5a6^cKO^* mutants (**D**) were comparable to control mice. (**E**–**L**) Quantification of ECG traces from 8-week mice (**E**–**H**) and 20-week mice (**I**–**L**) on a normal diet, showed a significant decrease in heart rate, increase in PR interval, widening of the QRS complex (20-weeks only), and loss of the J wave amplitude in *Slc5a6^cKO^* mutants. No differences were observed in the vitamin-supplemented *Slc5a6^cKO^* mice compared to vitamin-supplemented control mice (8 weeks: Con *n* = 13, cKO *n* = 12, ConV *n* = 12, cKOV *n* = 10; 20 weeks: Con *n* = 10, cKO *n* = 14, ConV *n* = 12, cKOV *n* = 10). Con, control; cKO, *Slc5a6^cKO^*; ConV, vitamin-supplemented control; cKOV, vitamin-supplemented *Slc5a6^cKO^*. Data are represented as mean ± SEM. ns, nonsignificant. **P < 0.01, ***P < 0.001, ****P < 0.0001 by 1-way ANOVA with Bonferroni’s correction for multiple comparisons (**E** and **J**) or nonparametric Kruskal-Wallis test with Dunn’s correction for multiple comparisons (**F**–**I**, **K**, and **L**).

**Figure 5:**
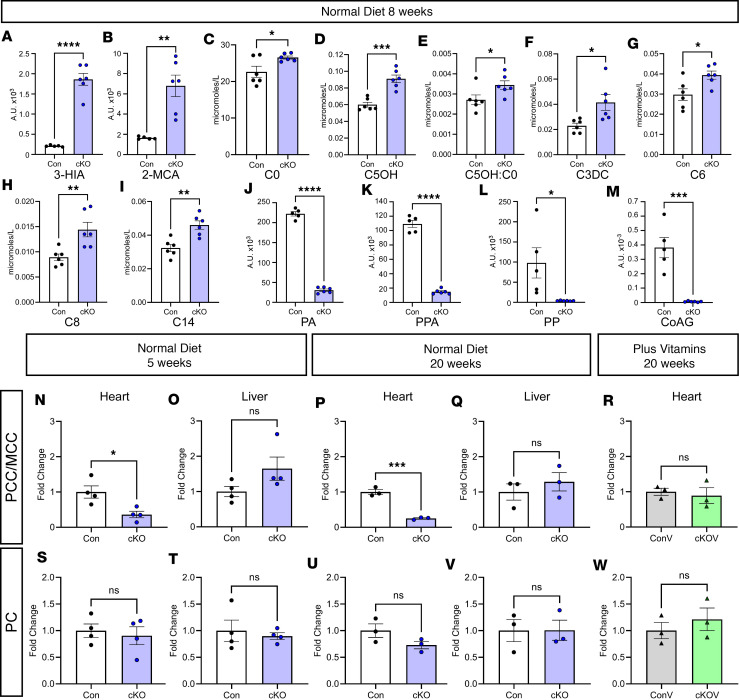
Reduced vitamin transport in hearts from *Slc5a6^cKO^* hearts. (**A**–**M**) Metabolic analysis revealed significant increases in 3-HIA and 2-MCA in *Slc5a6^cKO^* mutants (*n* = 6) compared with controls (*n* = 5) (**A** and **B**). (**C**–**I**) Acylcarnitine analysis of plasma showed significant increases in C0, C5OH, C5OH/C0 ratio, C3DC, C6, C8, and C14 in *Slc5a6^cKO^* mutants (*n* = 6) compared with controls (*n* = 5). (**J**–**M**) Reduced pantothenic acid uptake was observed in *Slc5a6^cKO^* mutants (**J**) as well as a significant decrease in PA, PPA, PP, and CoAG. (**N**–**W**) In heart protein samples (*n* = 3–4 for each genotype), a decrease in PCC/MCC biotinylation was observed in *Slc5a6^cKO^* mutants at 5 and 20 weeks (**N** and **P**), with no corresponding change in liver samples (**O** and **Q**). In vitamin-supplemented *Slc5a6^cKO^* mutants, PCC/MCC biotinylation levels in the hearts were comparable to controls (**R**). No change in PC was observed in the hearts or livers (**S**–**W**). Data are represented as mean ± SEM. ns, nonsignificant. *P < 0.05, **P < 0.01, ***P < 0.001, ****P < 0.0001 by unpaired *t* test for 2-sample comparisons. 3-HIA, 3-hydroxyisovaleric acid; 2-MCA, 2-methylcitric acid; C0, free carnitine; C5OH, 3-hydroxyisovaleryl-carnitine; C3DC, malonylcarnitine; C6, hexanoylcarnitine; C8, octanoylcarnitine; C14, tetradecanoylcarnitine; PA, pantothenic acid; PPA, phosphopantothenic acid; PP, phosphopantetheine; CoAG, CoA-glutathione; MCC, 3-methylcrotonyl-CoA carboxylase; PCC, propionyl-CoA carboxylase; PC, pyruvate carboxylase; Con, control; cKO, *Slc5a6^cKO^*; ConV, vitamin-supplemented control; cKOV, vitamin-supplemented *Slc5a6^cKO^*.

**Figure 6:**
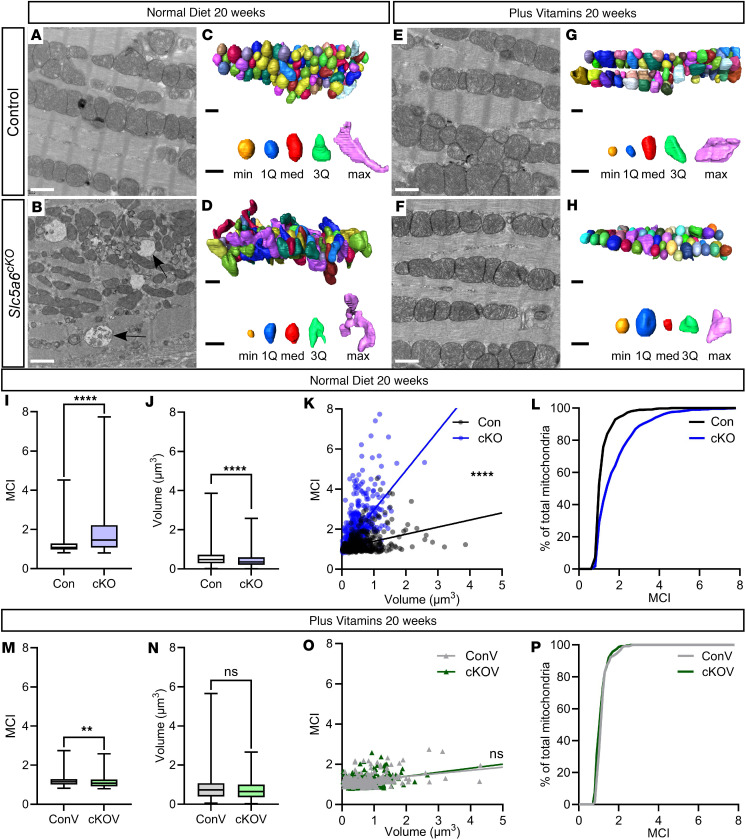
Increased mitochondrial complexity following loss of *Slc5a6* is prevented by vitamin supplementation. (**A**–**H**) Mitochondria imaging at 20 weeks by TEM (**A**, **B**, **E**, and **F**) and 3D reconstructions (**C**, **D**, **G**, and **H**). Mitochondria in controls, vitamin-supplemented controls, and vitamin-supplemented *Slc5a6^cKO^* hearts were all comparable (**A**, **C**, and **E**–**H**), whereas mitochondria in *Slc5a6^cKO^* hearts (**B** and **D**) were unevenly arranged and irregularly shaped with evidence of mitochondrial degradation (arrows in **B**). Representative individual mitochondria of the minimum (min), first quartile (1Q), median (med), third quartile (3Q), and maximum (max) MCI are shown (total number of mitochondria analyzed: Con = 468, cKO = 530*,* ConV = 241 and cKOV = 240). (**I**–**L**) The MCI was significantly increased in 20-week *Slc5a6^cKO^* mice on a normal diet (**I**) and the mitochondrial volume was significantly decreased (**J**). Bivariate analysis of MCI versus volume shows a significant difference in the regression line (**K**) and the cumulative frequency distribution curves do not overlap (**L**). (**M**–**P**) In vitamin-supplemented *Slc5a6^cKO^* mice, the MCI was significantly reduced (**M**) and there was no difference in mitochondrial volume (**N**) and complexity (**O** and **P**). Data are represented as mean ± SEM. ns, nonsignificant. **P < 0.01, ****P < 0.0001 by nonparametric Mann-Whitney test for two sample comparisons (**I**, **J**, **M**, and **N**) and the slopes of the regression lines were compared using an 1-way Analysis of Covariance (ANCOVA) (**K** and **O**). Con, control; cKO, *Slc5a6^cKO^*; ConV, vitamin-supplemented control; cKOV, vitamin-supplemented *Slc5a6^cKO^*. Scale bars: 500 nm (**A**, **B**, **E**, and **F**) and 1 μm (**C**, **D**, **G**, and **H**).

**Figure 7:**
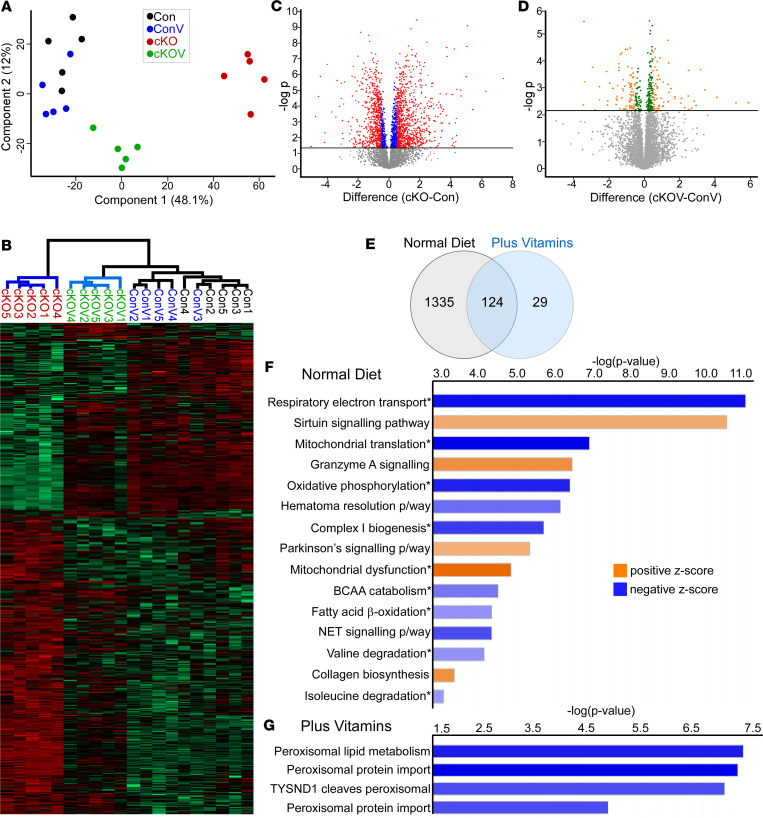
Proteomic analysis revealed altered metabolism and increased fibrosis correlate with early indicators of cardiomyopathy in *Slc5a6^cKO^* mice. (**A**–**E**) Proteomic analysis at 8 weeks. PCA plot (**A**) and heatmap (**B**) illustrate that the proteome of the *Slc5a6^cKO^* mutants was distinct from the 3 other groups (control, vitamin-supplemented control, and vitamin-supplemented *Slc5a6^cKO^* hearts; *n* = 5 per group). (**C** and **D**) Volcano plots showing proteins with a significant (*q* < 0.05) fold change. (**C**) Under a normal diet, 1459 proteins with a fold change of ≤ –0.5 and ≥ 0.5 (red dots) and 478 proteins with a fold change of > –0.5 and < 0.5 (blue dots) were seen. (**D**) With vitamin supplementation, only 153 proteins with a fold change of ≤ –0.5 and ≥ 0.5 (orange dots) and 179 proteins with a fold change of > –0.5 and < 0.5 (green dots) were seen. (**E**) Venn diagram illustrating 124 of the proteins with a fold change of ≤ –0.5 and ≥ 0.5 remained significantly differentially expressed following vitamin supplementation. (**F** and **G**) IPA analysis of up- and downregulated proteins. For the mice on a normal diet, the majority of the enriched downregulated pathways in *Slc5a6^cKO^* mutant mice are linked to different components of the energy metabolism pathways in mitochondria (shown by asterisks in **F**) (**F**). In comparison, vitamin-supplemented *Slc5a6^cKO^* mutant mice only had downregulation of peroxisome-related pathways (**G**).

**Figure 8:**
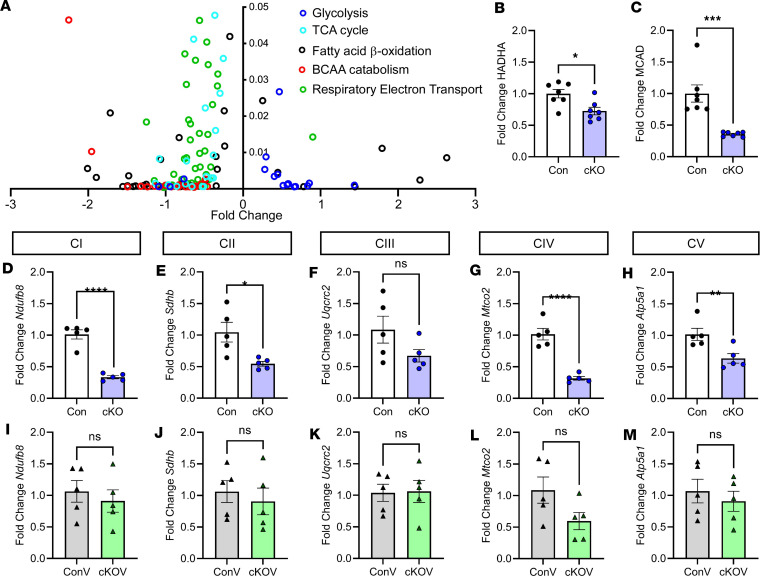
Broad suppression of mitochondrial energy metabolism pathways and electron transport chain components in *Slc5a6^cKO^* hearts. (**A**) Summary of 5 different pathways involved in energy metabolism identified in the proteomics analysis of 8-week hearts. The majority of proteins in the TCA cycle, fatty acid β-oxidation, branched-chain amino acid (BCAA) catabolism, and electron transport chain (ETC) are downregulated in *Slc5a6^cKO^* mutants, with an increase in proteins associated with glycolysis. (**B** and **C**) Quantification of Western blot analyses showed reduced abundance of representative β-oxidation enzymes HADHA and MCAD in 5-week *Slc5a6^cKO^* hearts compared with controls. (**D**–**M**) qPCR analysis of genes representing a subunit from each complex (CI–CV) of the ETC (CI: *Ndufb8*, CII: *Sdhb*, CIII: *Uqcrc2*, CIV: *Mtco2* and CV: *Atp5a1*) in 8-week hearts. There was a significant downregulation in CI, CII, CIV, and CV in *Slc5a6^cKO^* hearts (**D**–**H**) and no corresponding decrease in vitamin-treated hearts (**I**–**M**) (*n* = 5 hearts for each genotype). Data are represented as mean ± SEM. ns, nonsignificant. *P < 0.05, **P < 0.01, ***P < 0.001, ****P < 0.0001 by unpaired *t* test for 2 sample comparisons. Con, control; cKO, *Slc5a6^cKO^*; ConV, vitamin-supplemented control; cKOV, vitamin-supplemented *Slc5a6^cKO^*.

**Figure 9:**
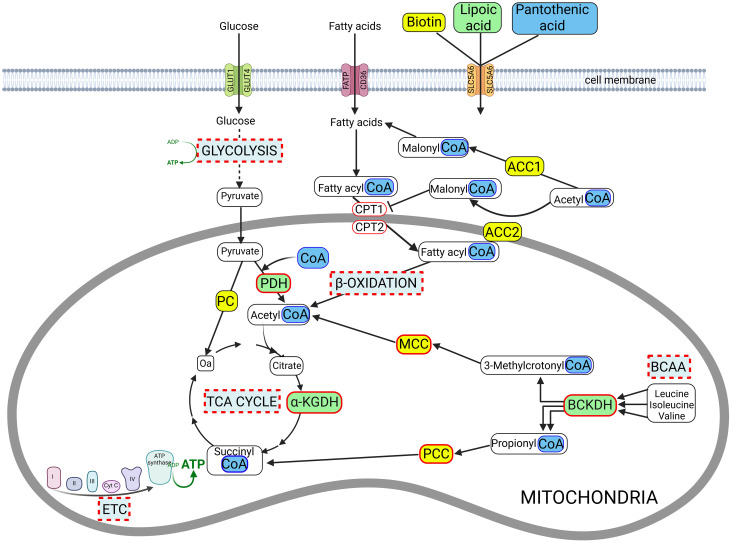
Integrated model of altered cardiac energy metabolism pathways in *Slc5a6^cKO^* hearts. Schematic overview integrating proteomic and metabolomic findings, illustrating affected energy metabolism pathways and factors, in *Slc5a6^cKO^* hearts (outlined in red). Vitamins and their role in the pathways are color coded: biotin, yellow; lipoic acid, green; and pantothenic acid, blue.

**Table 1:**
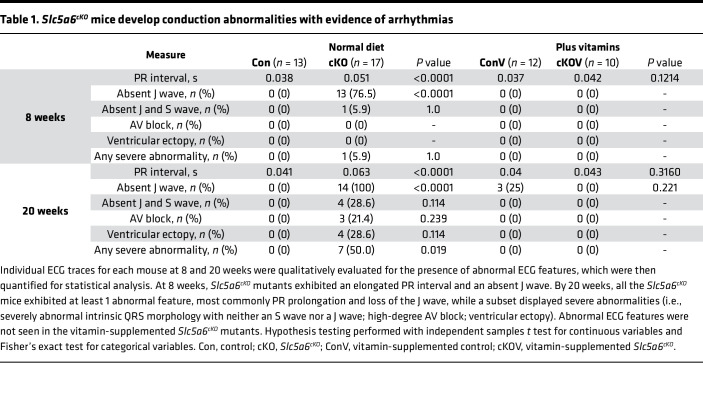
*Slc5a6^cKO^* mice develop conduction abnormalities with evidence of arrhythmias

## Version 1 06/02/2026

In-Press Preview

## Version 2 07/22/2026

Electronic publication
