# Supplementary material for: A Slc5a6-deficient mouse model reveals metabolically driven cardiomyopathy with therapeutic potential for vitamin-based intervention
Source: JCI Insight. 2026 Jun 2;11(14):e200381. doi: 10.1172/jci.insight.200381 (PMC13461167; doi:10.1172/jci.insight.200381)
Supplement: Supplementary file 1 [file jciinsight-11-200381-s104.pdf]

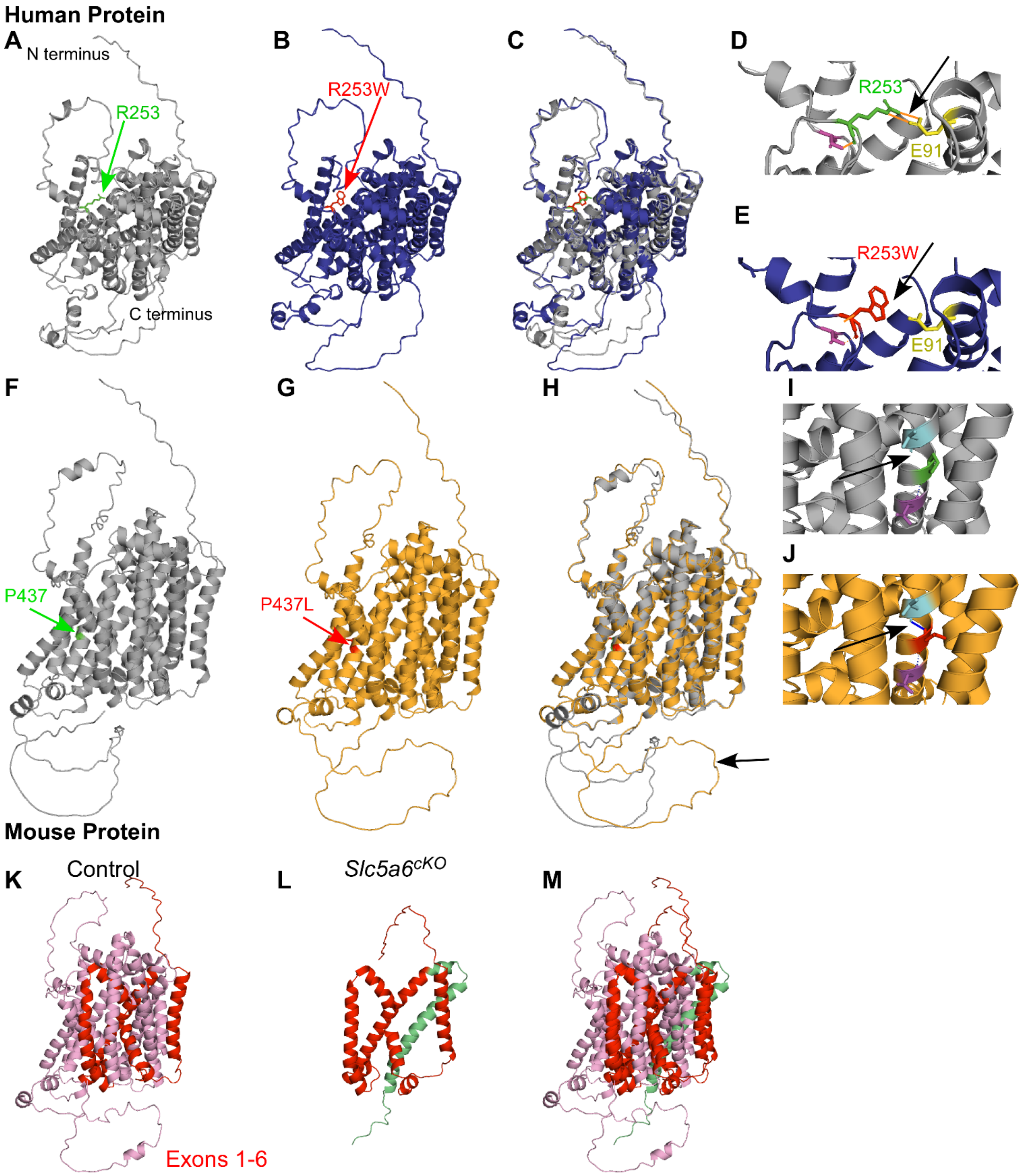

Supplemental Figure 1. Structural modelling of human SLC5A6 protein variants and truncation in the mouse *Slc5a6^{cKO}* model

(**A-E**) Protein modelling of SMVT with R253 in the wildtype protein (**A**, grey model with R253 in green) and R253W in the mutant protein (**B**, blue model with R253W in red). The R253W mutation causes a salt bridge (orange) to be removed (**C**, black arrow in **D**,**E**). (**F-J**) 3D modelling of the SMVT protein highlighting P437 in the wildtype (**F**, grey model with P437 in green) and mutant (**G**, orange model with P437L in red) protein. The overall position of the intracellular loops was altered (arrow in **H**). Substitution with leucine retains the hydrogen bond with residue L441 (magenta) but introduced a novel polar bond with V434 (cyan; black arrow in **I**,**J**). (**K-M**) Mouse wildtype SLC5A6 protein (**K**,**M**, pink) is very similar structure to the human SLC5A6 protein. The predicted model of any potential residual SLC5A6 protein with exons 7-10 removed in the *Slc5a6^{cKO}* mouse is shown (**L**,**M**, green). Exons 1-6 (red) are predicted to be spliced to exon 11 (green) producing a truncated protein, which would be degraded.

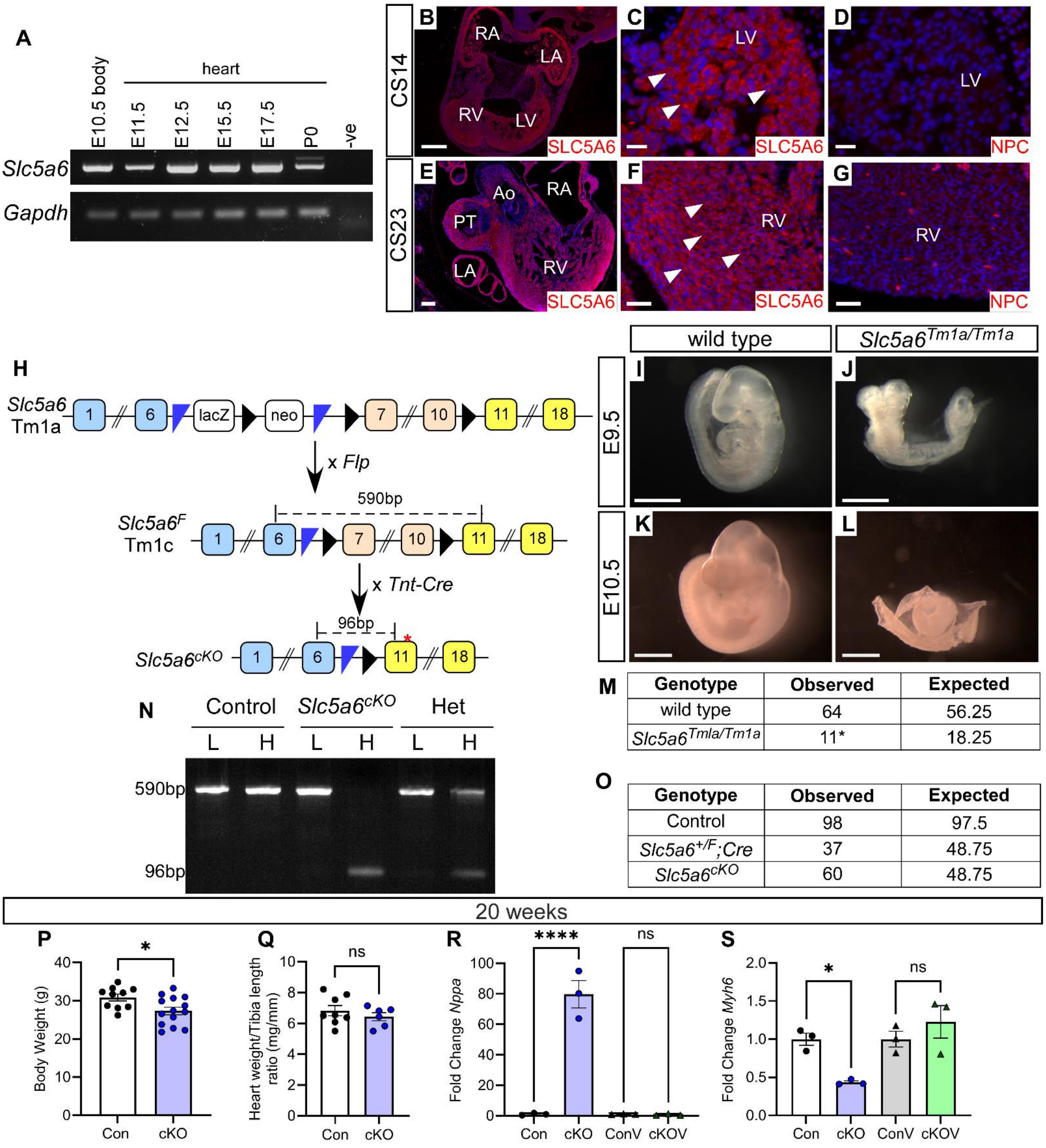

Supplemental Figure 2. Generation of *Slc5a6* transgenic mouse models

(**A**) RT-PCR showing *Slc5a6* expression in the developing heart (E11.5 to P0). (**B-G**) SLC5A6 antibody staining in human atrial and ventricular myocardium at CS14 and CS23 showing strong membrane staining (B,C,E,F arrowheads). No primary control (**D,G**) showed no specific staining. DAPI counterstain. (**H**) *Slc5a6* Tm1a allele contains a neomycin *lacZ* cassette flanked by *frt* sites (blue triangles) and *LoxP* sites (black triangles) flanking exons 7 to 10 of *Slc5a6*. Crossing with the *Flp* recombinase mouse generated the conditional *Slc5a6^{F} Tm1c* allele. Subsequent crossing with *Tnt-Cre* transgenic mice removed exons 7-10 within cardiomyocytes from E7.5, introducing a premature stop codon (red asterisk) in the SLC5A6 protein. (**I-M**) Homozygous *Slc5a6^{Tm1a}* embryos are embryonic lethal and are underrepresented between E9.5 to E15.5. (**N**) RT-PCR confirmed the deletion of exons 7-10 (96bp band) in *Slc5a6^{cKO}* hearts (H) and not in the limb (L) in E15.5 embryos. (**O**) *Slc5a6^{cKO}* mice were weaned at Mendelian ratios. (**P,Q**) At 20 weeks, *Slc5a6^{cKO}* showed

reduced body weight (control, n=10; *Slc5a6*^{cKO}, n=14) but unchanged heart weight/ tibia length (control, n=8; *Slc5a6*^{cKO}, n=6). (**R,S**) Cardiac dysfunction markers were altered in 20 week *Slc5a6*^{cKO} hearts; increased *Nppa* and decreased *Myh6* expression (n=3), but unchanged in 20-week vitamin-supplemented *Slc5a6*^{cKO} mice. Data are represented as mean ±SEM. ns, nonsignificant, *p < 0.05, **p < 0.01, ****p < 0.0001 by unpaired t-test for two sample comparisons or one-way ANOVA with Bonferroni correction for multiple comparisons. Con, Control; cKO, *Slc5a6*^{cKO}; ConV, vitamin-supplemented control; cKOV, vitamin-supplemented *Slc5a6*^{cKO}. RV, right ventricle; LV, left ventricle; RA, right atria; LA, left atria; PT, pulmonary trunk; Ao, aorta; NPC, no primary control. Scale bars B,E = 200µm, C,D,F,G = 20µm, I-L = 1mm.

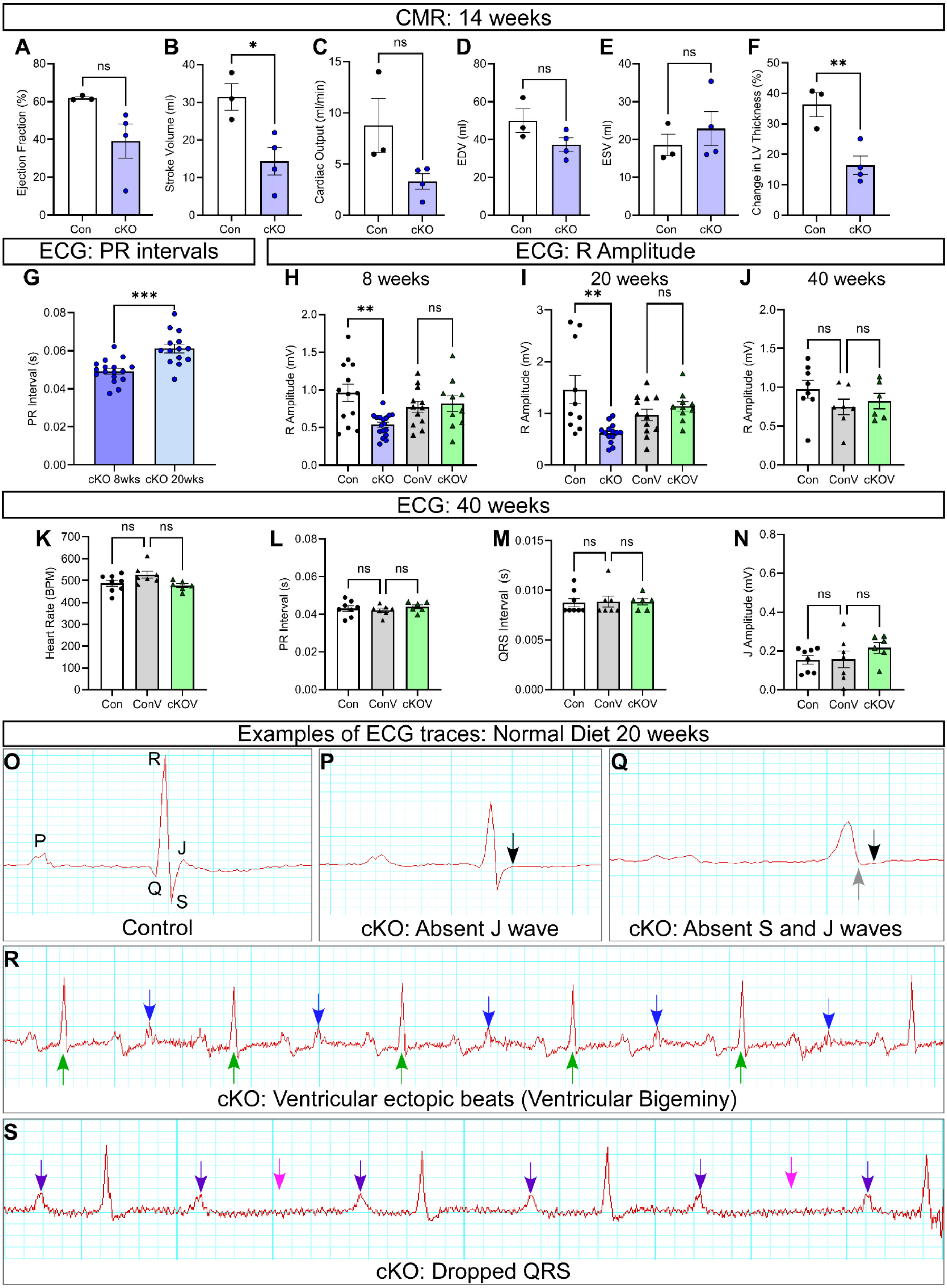
CMR: 14 weeks
A
Ejection Fraction (%)
Con
cKO
ns
B
Stroke Volume (ml)
*
C
Cardiac Output (ml/min)
D
EDV (ml)
E
ESV (ml)
F
Change in LV Thickness (%)
**
ECG: PR intervals
G
PR Interval (s)
***
cKO 8wks
cKO 20wks
ECG: R Amplitude
H
8 weeks
R Amplitude (mV)
ConV
cKOV
I
20 weeks
J
40 weeks
ECG: 40 weeks
K
Heart Rate (BPM)
L
PR Interval (s)
M
QRS Interval (s)
N
J Amplitude (mV)
Examples of ECG traces: Normal Diet 20 weeks
O
P
Q
R
S
J
Control
cKO: Absent J wave
cKO: Absent S and J waves
cKO: Ventricular ectopic beats (Ventricular Bigeminy)
cKO: Dropped QRS

Supplemental Figure 3. Further cardiac function analysis and evidence of conduction pathology and arrhythmias in *Slc5a6^{cKO}* mutant mice

(**A-F**) Cardiac magnetic resonance (CMR) imaging at 14 weeks, showing reduced stroke volume (**B**) and decreased % change in left ventricular (LV) wall thickness (**F**) in *Slc5a6^{cKO}* mutants (*n*=3) compared to controls (*n*=3). (**G**) ECG measurements from 8 weeks (*n*=17) to 20 weeks (*n*=14), revealed a significant increase in PR interval in *Slc5a6^{cKO}* mutants. (**H,I**) Quantification of ECG traces from 8-week mice (**H**) and 20-week mice (**I**) on a normal diet, showed a significant decrease in R amplitude in *Slc5a6^{cKO}* mutants with no differences in the vitamin-supplemented *Slc5a6^{cKO}* mice compared to control mice. At 40 weeks (**J-N**) no significant differences were observed in the control mice on a normal diet and vitamin-supplemented control and *Slc5a6^{cKO}* mice. (8-weeks: Con *n*=13, cKO *n*=12, ConV *n*=12, cKOV *n*=10; 20-weeks: Con *n*=10, cKO *n*=14, ConV *n*=12, cKOV *n*=10; 40-weeks: Con *n*=8, ConV *n*=7, cKOV *n*=6). (**O-S**) Representative ECG traces from 20-week mice on a normal diet. The control trace has a clear normal P, QRS, and J morphology (**O**), whereas all *Slc5a6^{cKO}* mutant mice have absent J waves (black arrow in **P**) and 28.6% also have absent S waves (grey arrow in **Q**). Ventricular ectopic beats (blue arrows in **R**) were evident in 28.6% of *Slc5a6^{cKO}* mutant mice. An example of ventricular bigeminy is shown where there was a ventricular ectopic beat after each sinus beat (green arrows in **R**). Evidence of high-degree atrioventricular block was also observed in 21.4% of *Slc5a6^{cKO}* mutant mice, with dropped QRS complexes (pink arrows in **S**) after the P waves (purple arrows in **S**). Con, control; cKO, *Slc5a6^{cKO}*; ConV, vitamin-supplemented control; cKOV, vitamin-supplemented *Slc5a6^{cKO}*. Data are represented as mean ±SEM. ns = nonsignificant, *$p < 0.05$, **$p < 0.01$, ***$p < 0.001$, by unpaired t-test for two sample comparisons, one-way ANOVA with Bonferroni correction for multiple comparisons or nonparametric Kruskal-Wallis test with Dunn's correction for multiple comparisons.

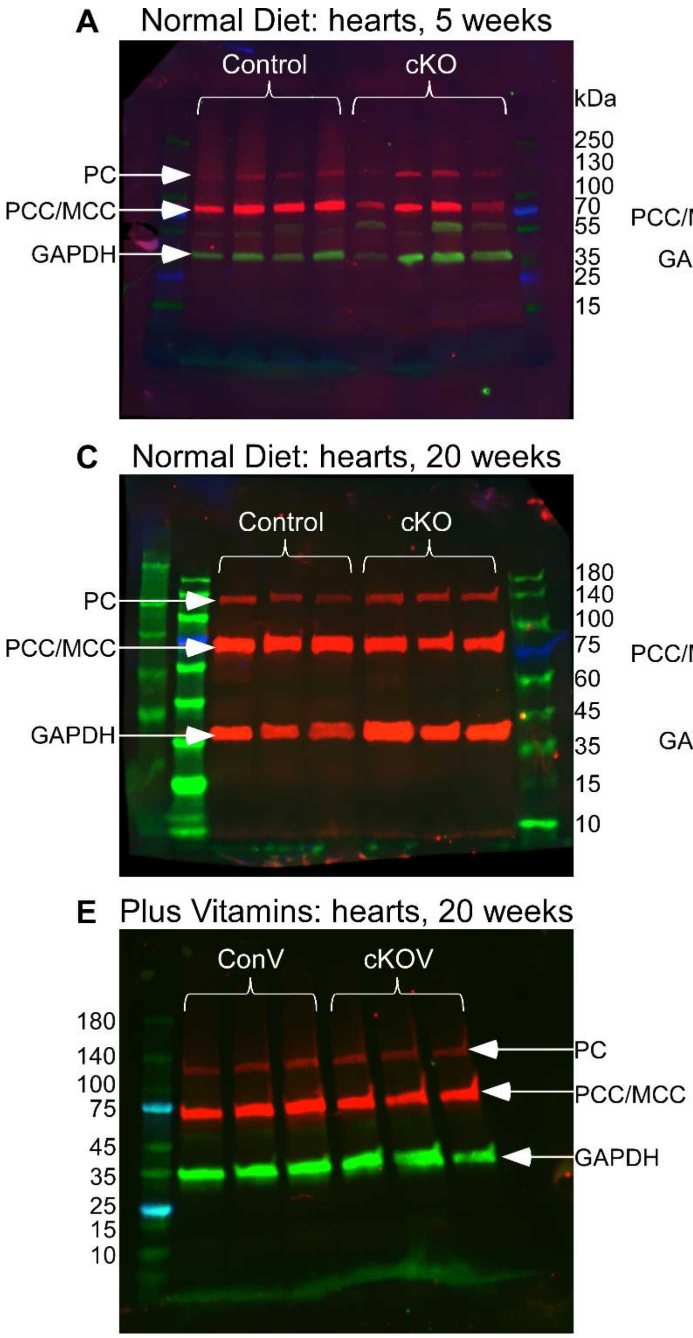

Supplemental Figure 4. Measurement of protein biotinylation

Western blots were probed with Streptavidin and GAPDH antibodies. (**A,B**) Western blots of 5-week hearts and livers on a normal diet (n=4). (**C-E**) Western blots from 20-week hearts and livers on a normal diet and 20-week hearts from vitamin-supplemented diets (n=3). PCC and MCC comigrate at 75kDa, PC at 130kDa and GAPDH at 37kDa. ACC1/2 (265kDa) was not visible on these blots. MCC, 3-methylcrotonyl CoA carboxylase; PCC, propionyl-CoA carboxylase; PC, pyruvate carboxylase; Con, Control; cKO, *Slc5a6*^{cKO}; ConV, vitamin-supplemented control; cKOV, vitamin-supplemented *Slc5a6*^{cKO}.

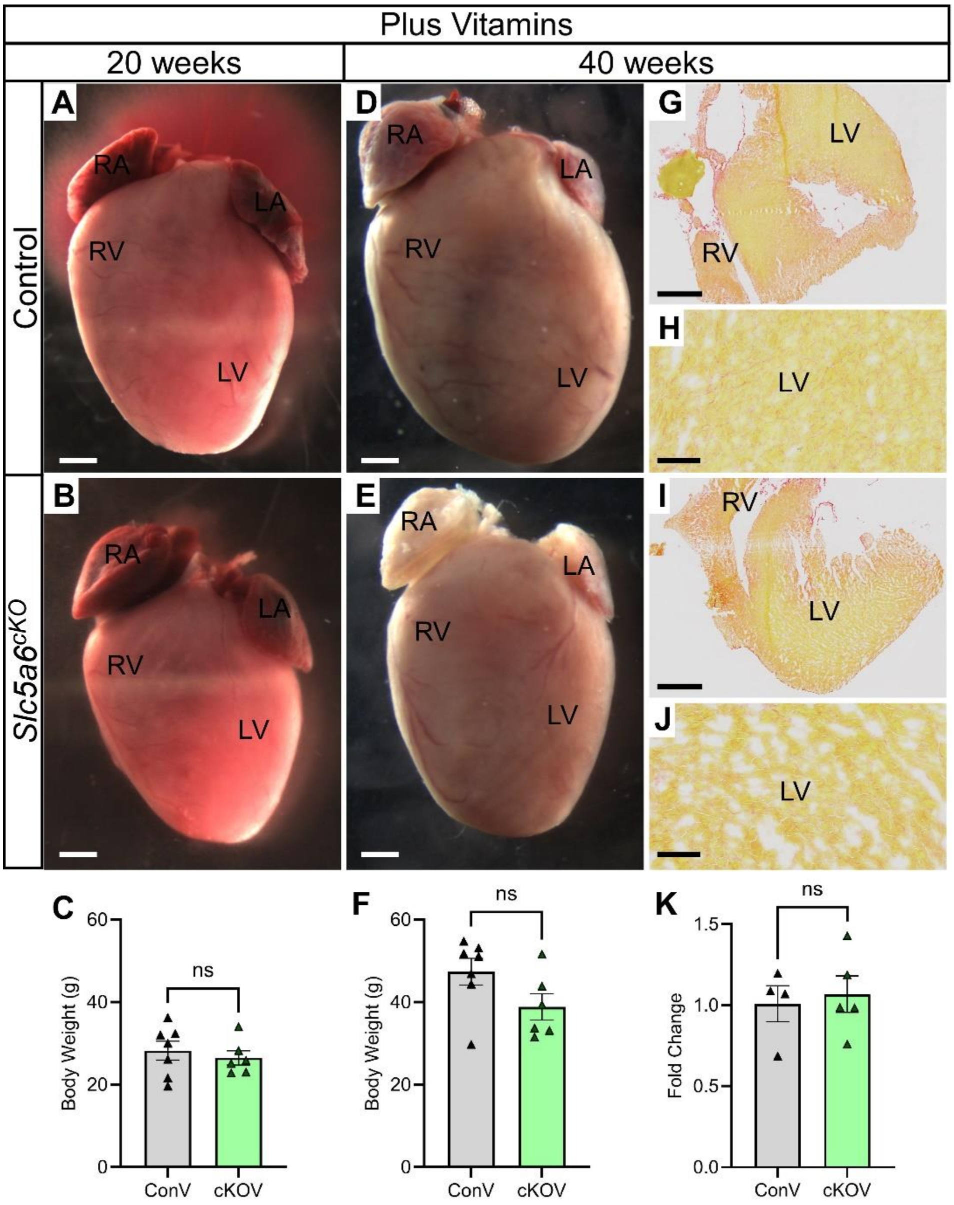

Supplemental Figure 5. Vitamin supplementation prevents cardiomyopathy

Hearts were dissected from 20-week (**A,B**) and 40-week (**D,E**) vitamin-supplemented mice. Control and *Slc5a6^{cKO}* hearts were comparable with no evidence of enlargement. No difference in body weight was observed at 20 weeks (**C**) or 40 weeks (**F**; ConV *n*=7, cKOV *n*=6). (**G-K**) Picrosirius Red staining of heart sections from 40-week hearts, showed there was no significant increase in fibrosis in the vitamin-supplemented *Slc5a6^{cKO}* mutant hearts. Data are represented as mean ±SEM. ns, nonsignificant, by unpaired t-test for two sample comparisons. RV, right ventricle; LV, left ventricle; RA, right atria; LA, left atria; ConV, vitamin-supplemented control; cKOV, vitamin-supplemented *Slc5a6^{cKO}*. Scale bars A,B,D,E,G,I = 1mm, H,J = 100µm.

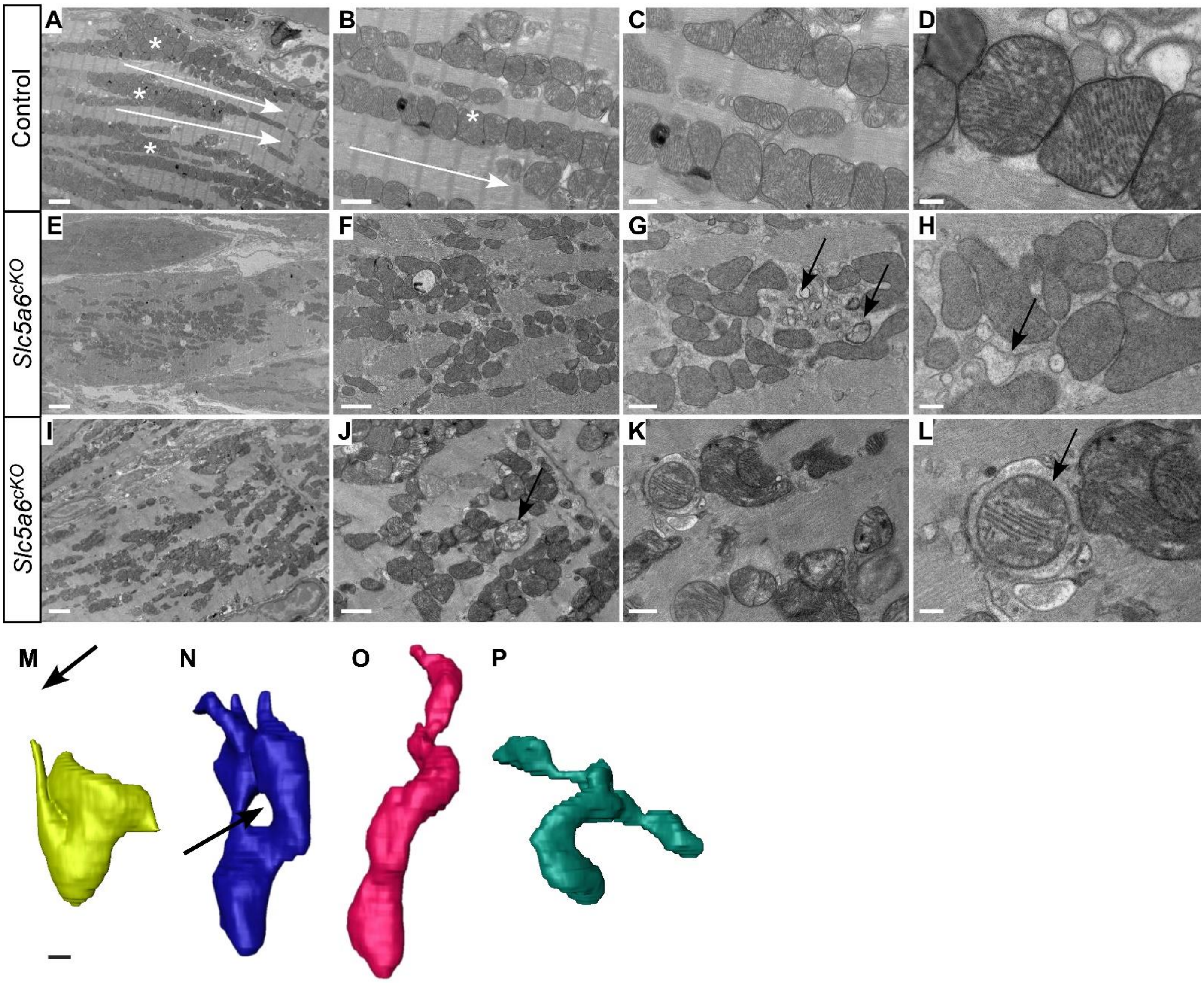

Supplemental Figure 6. Further examples of abnormal mitochondrial morphology in 20-week *Slc5a6^{cKO}* hearts

(**A-D**) Representative TEM images of a control 20-week heart showing the regular arrangement of clusters of mitochondria (white asterisks), parallel to sarcomeres (white arrows). (**E-L**) Representative TEM images of *Slc5a6^{cKO}* mice. The mitochondria do not form organised clusters and are randomly dispersed, with irregular sizes and shapes, and evidence of fragmented and damaged mitochondria (black arrows). (**M-P**) Examples of the different mitochondrial morphologies observed in the *Slc5a6^{cKO}* mice. Mitochondria with nanotunnels (**M**), donut shaped (**N**), elongated (**O**) and branched (**P**) mitochondria. Scale bars A,E,I = 2µm, B,F,J = 1µm, C,G,K = 500nm, D,H,L = 200nm, M-P = 0.3µm.

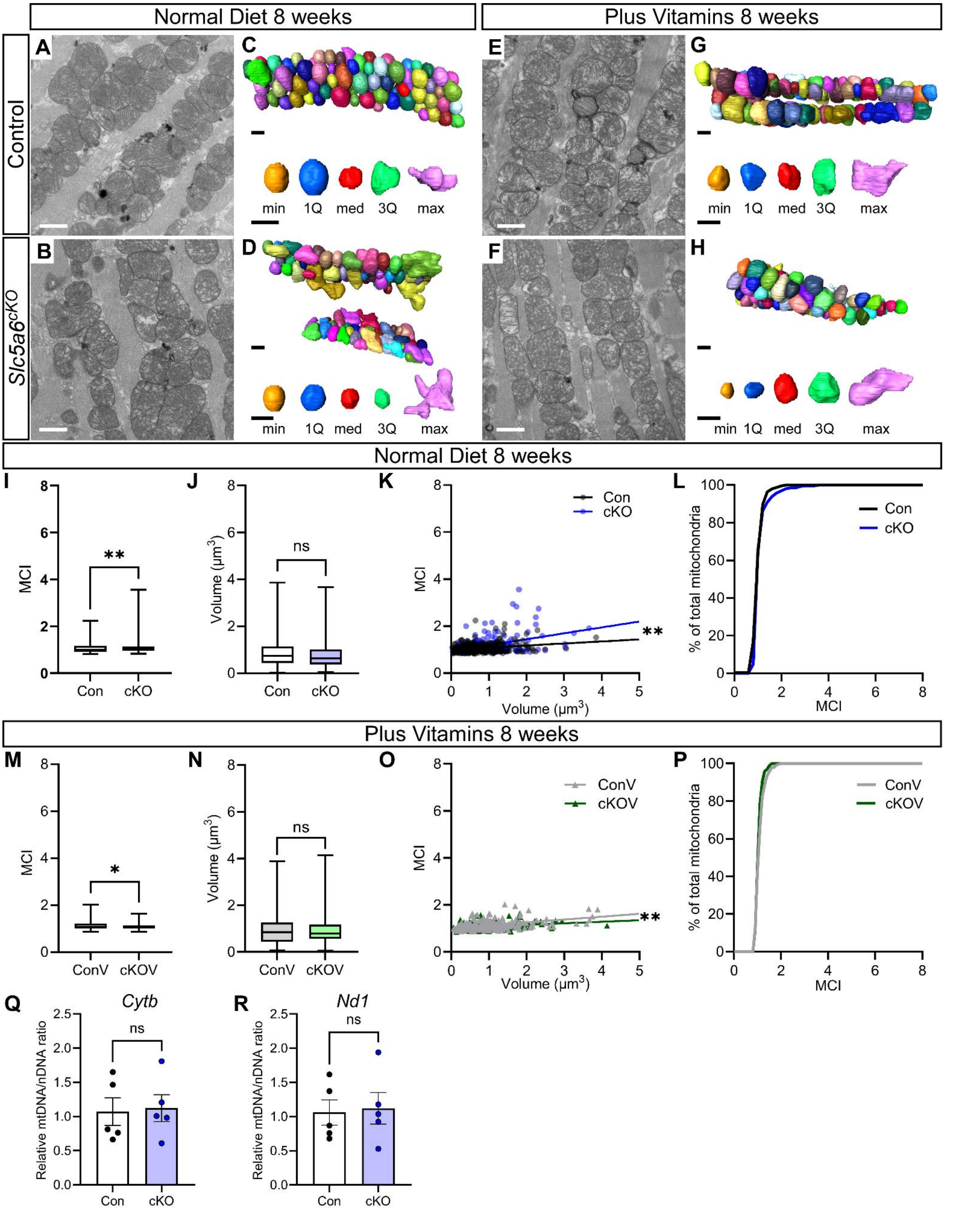

Supplemental Figure 7. Subtle changes in mitochondrial morphology seen in 8-week *Slc5a6^{cKO}* mice

(**A-H**) Cardiac mitochondria images from normal diet and vitamin-supplemented hearts from 8-week mice (*n*=3). No obvious differences were seen in TEM images (**A**,**B**,**E**,**F**). Representative 3D reconstructions of mitochondrial clusters are shown (**C**,**D**,**G**,**H**). The MCI was calculated for each mitochondria and representative individual mitochondria of the minimum (min), first quartile (1Q), median (med), third quartile (3Q) and maximum (max) MCI are shown (total number

of mitochondria analysed: Con=251, cKO=264, ConV=240 and cKOV=261). (**I-L**) For 8-week mice on a normal diet the MCI of all of the mitochondria in the *Slc5a6*^{cKO} hearts was significantly increased, but there was no difference in volume. Bivariate analysis of MCI:volume showed a significant difference in the regression line (**K**) and the cumulative frequency distribution show partial overlap (**L**). (**M-P**) Vitamin supplementation reduced the MCI in *Slc5a6*^{cKO} mice and the complexity of the mitochondria. (**Q**,**R**) Mitochondria DNA copy number was quantified by determining the mtDNA/nDNA ratio in 8-week hearts on a normal diet (n=5), using two different mtDNA genes (*Cytb* and *Nd1*). There was no significant difference between control and *Slc5a6*^{cKO} hearts. Data are represented as mean ±SEM. ns = nonsignificant, *p < 0.05, **p < 0.01, by nonparametric Mann-Whitney test or unpaired t-test. Con, control; cKO, *Slc5a6*^{cKO}*;* ConV, vitamin-supplemented control; cKOV, vitamin-supplemented *Slc5a6*^{cKO}. Scale bars A,B,E,F = 500nm, C,D,G,H = 1µm.

A Normal Diet 5 weeks: HADHA

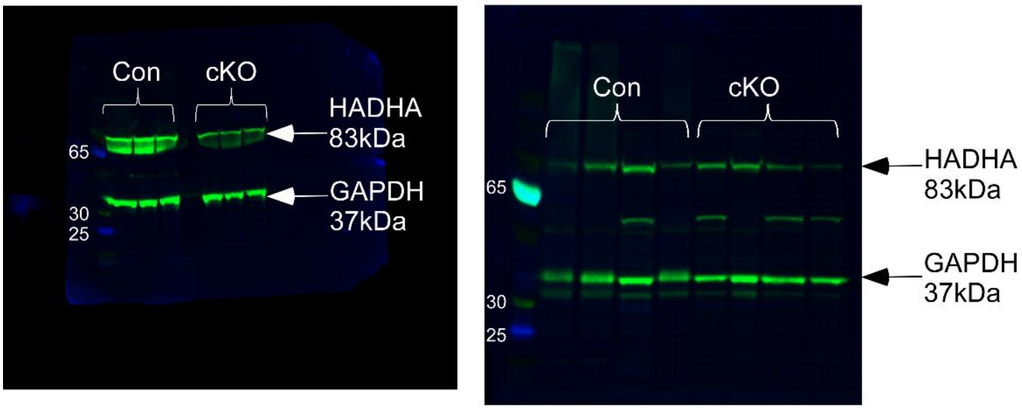

B Normal Diet 5 weeks: MCAD

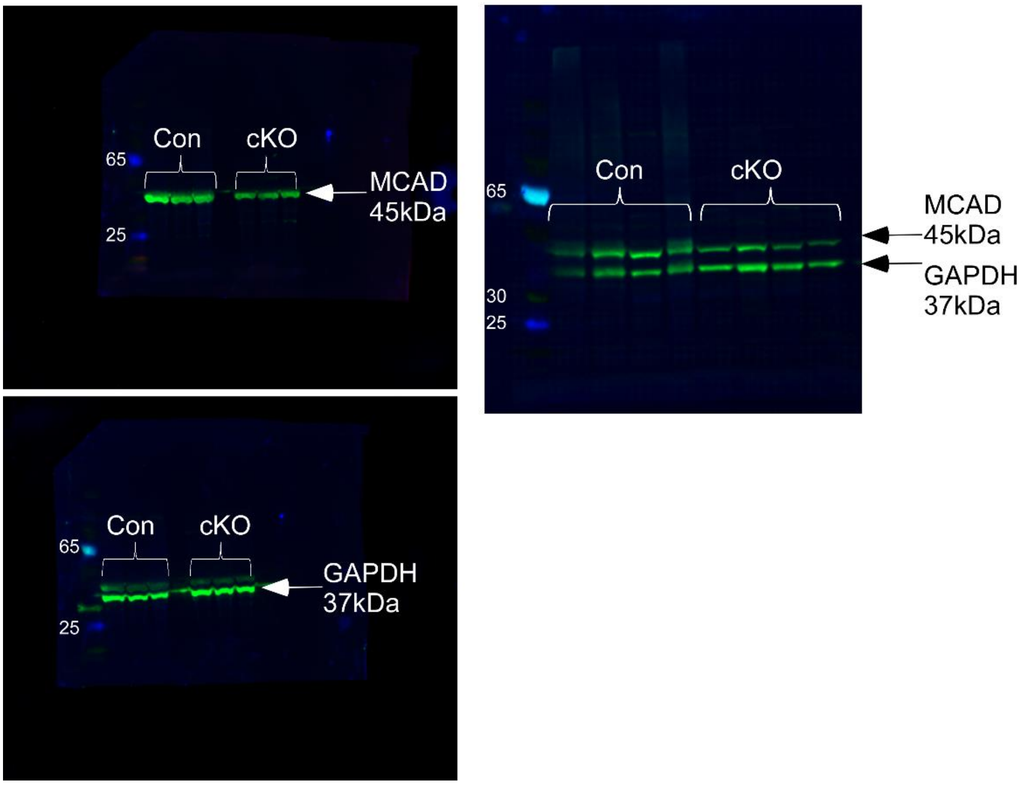

Supplemental Figure 8. Expression of β-oxidation enzymes, HADHA and MCAD, were decreased in *Slc5a6*^{cKO} mutant hearts

Western blots of 5-week hearts on a normal diet (*n*=7) were probed with HADHA (**A**) or MCAD (**B**) antibodies. GAPDH was used as a loading control. Brackets denote biological replicates within each group. HADHA is 83kDa, MCAD is ~45kDa, GAPDH is 37kDa. Con, Control; cKO, *Slc5a6*^{cKO}.

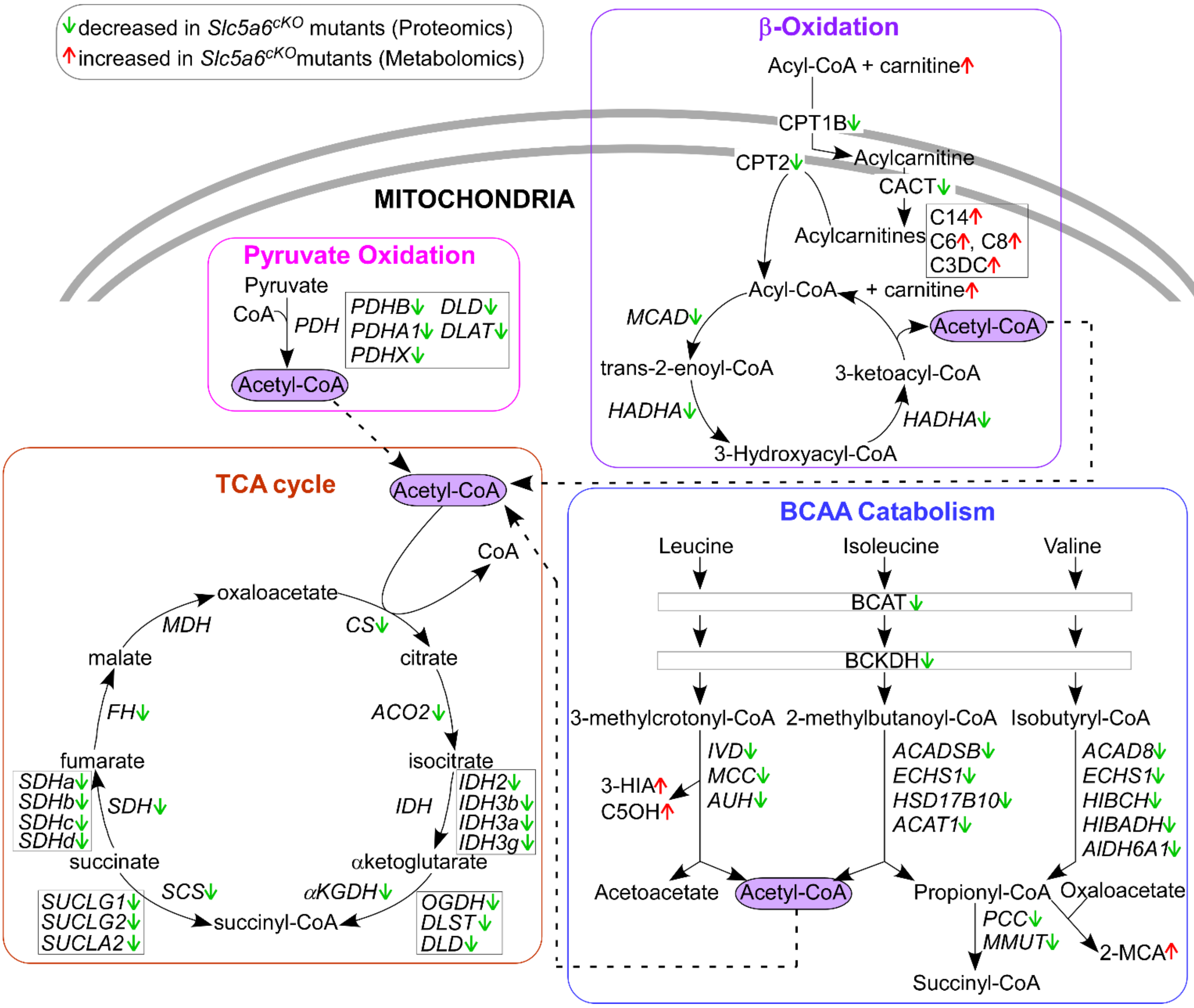

Supplemental Figure 9. Coordinated disruption of mitochondrial energy metabolism pathways in *Slc5a6*^{cKO} hearts

Schematic overview integrating proteomic and metabolomic findings to illustrate coordinated alterations in mitochondrial energy metabolism in 8-week *Slc5a6*^{cKO} hearts. Green downward arrows indicate proteins that are downregulated based on proteomic analysis, and red upward arrows denote metabolites increased based on metabolomic analysis. Pyruvate oxidation is impaired by coordinated downregulation of all components of the pyruvate dehydrogenase (PDH) complex, a lipoic-acid- and coenzyme-A-dependent pathway that supplies acetyl-CoA. β-oxidation is disrupted at multiple steps, with reduced expression of fatty-acid transport and oxidation enzymes accompanied by accumulation of medium- and long-chain acylcarnitines. The TCA cycle exhibits coordinated downregulation of several enzymatic components, suggesting reduced cycle capacity to oxidise acetyl-CoA. Branched-chain amino acid (BCAA) catabolism is similarly suppressed, with decreased expression of enzymes involved in leucine, isoleucine and valine oxidation and evidence of propionyl-CoA overflow, including accumulation of C5OH, 3-hydroxy-isovaleric acid (3-HIA) and 2-methylcitrate (2-MCA).

CMR Data

14 weeks mean ±SEM	Con	cKO	*Con vs cKO p value*
**Ejection Fraction (%)**	61.79±0.74	39.05 ±9.03	*0.0869*
**Stroke Volume(ml)**	31.42 ±3.6	14.32 ±3.64	*0.0226*
**Cardiac Output (ml/min)**	8.77 ±2.62	3.32 ±0.75	*0.0692*
**EDV (ml)**	49.97 ±6.23	37.2 ±3.57	*0.1153*
**ESV (ml)**	18.56 ±2.87	22.88 ±4.47	*0.4902*

20 weeks mean ±SEM	Con	cKO	*Con vs cKO p value*	ConV	cKOV
**Ejection Fraction (%)**	60.94 ±8.05	28.8 ±6.145	*0.0093*	61.74 ±1.3	64.8 ±0.14
**Stroke Volume(ml)**	34.37 ±4.20	16.09 ±3.37	*0.0065*	29.2 ±2.48	26.75 ±1.69
**Cardiac Output (ml/min)**	15.82 ±1.89	6.765 ±1.11	*0.0024*	11.73 ±0.11	9.38 ±0.14
**EDV (ml)**	57.56 ±4.33	55.92 ±2.9	*0.7758*	47.4 ±5.01	41.26 ±2.53
**ESV (ml)**	23.19 ±5.64	39.83 ±3.834	*0.0367*	18.2 ±2.53	14.52 ±0.83

ECG Data

8 weeks mean ±SEM	Con	cKO	*Con vs cKO p value*	ConV	cKOV	*ConV vs cKOV p value*
**Heart rate (bpm)**	503 ±12.94	434.2 ±6.88	*0.0004*	510.9 ±10.63	478.1 ±19.86	*0.2979*
**PR interval (s)**	0.038 ±<0.001	0.051 ±0.001	*<0.0001*	0.037 ±<0.001	0.042 <0.001	*0.1214*
**QRS complex (s)**	0.009 ±<0.001	0.01 ±<0.01	*0.7796*	0.008 ±<0.001	0.01 ±<0.001	*0.1773*
**J Amplitude (mV)**	0.128 ±0.178	0.003 ±0.009	*0.0003*	0.118 ±0.029	0.184 ±0.023	*0.6746*
**R Amplitude(mV)**	0.962 ±0.113	0.538 ±0.036	*0.0013*	0.77 ±0.074	0.816 ±0.104	*0.9823*

20 weeks mean ±SEM	Con	cKO	*Con vs cKO p value*	ConV	cKOV	*ConV vs cKOV p value*
**Heart rate (bpm)**	501.3 ±10.72	409.7 ±23.86	*0.0024*	508.5 ±8.471	478.5 ±8.152	*0.4062*
**PR interval (s)**	0.041 ±<0.001	0.063 ±0.002	*<0.0001*	0.04 ±<0.001	0.043 ±<0.001	*0.3160*
**QRS complex (s)**	0.009 ±<0.001	0.123 ±<0.001	*0.0046*	0.008 ±<0.001	0.008 ±<0.001	*>0.9999*
**J Amplitude (mV)**	0.236 ±0.053	-0.017 ±0.013	*0.0007*	0.147 ±0.028	0.298 ±0.044	*0.2957*
**R Amplitude(mV)**	1.462 ±0.272	0.622 ±0.05	*0.0032*	0.973 ±0.11	1.138 ±0.092	*>0.9999*

40 weeks mean ±SEM	Con	ConV	*Con vs ConV p value*	cKOV	*ConV vs cKOV p value*
**Heart rate (bpm)**	487.7 ±13.78	526.8 ±16.11	*0.0961*	476.9 ±9.173	*0.8131*
**PR interval (s)**	0.043 ±0.001	0.042 ±0.001	*0.8688*	0.044 ±0.001	*0.6059*
**QRS complex (s)**	0.009 ±<0.001	0.009 ±<0.001	*>0.9999*	0.009 ±<0.001	*>0.9999*
**J Amplitude (mV)**	0.154 ±0.022	0.159 ±0.033	*>0.9999*	0.216 ±0.028	*0.3475*
**R Amplitude(mV)**	0.975 ±0.116	0.747 ±0.1	*0.2938*	0.823 ±0.1	*0.8808*

Supplemental Table 1. Data from cardiac function experiments

The mean ±SEM and *p* values for the CMR and ECG analyses shown in Figures 3 and 4 and Supplemental Figure 3. Con, Control; cKO, *Slc5a6*^{cKO}; ConV, vitamin-supplemented control; cKOV, vitamin-supplemented *Slc5a6*^{cKO}.

	Con (mean ±SEM)	cKO (mean ±SEM)	*p* value
3-hydroxyisovaleric acid (3-HIA)	209.4 ±8.88	1862 ±147.6	<0.0001
2-methylcitric acid (2-MCA)	1643 ±60.7	6794 ±1056	0.002
Free carnitine (C0)	22.64 ±1.514	26.55 ±0.4146	0.049
Hydroxyisovaleryl-carnitine (C5OH)	0.06 ±0.003	0.09 ±0.005	<0.001
C5OH:C0	0.003 ±0.0002	0.003 ±0.0002	0.044
Malonylcarnitine (C3DC)	0.023 ±0.002	0.042 ±0.006	0.03
Hexanoylcarnitine (C6)	0.03 ±0.003	0.04 ±0.002	0.022
Octanoylcarnitine (C8)	0.007 ±0.0007	0.014 ±0.001	0.011
Tetradecanoylcarnitine (C14)	0.032 ±0.002	0.046 ±0.003	0.002
Pantothenic acid (PA)	$22.2x10^4$ $±5.5x10^3$	$3.06x10^4$ $±2.9x10^3$	<0.0001
Phosphopantothenic acid (PPA)	$10.9x10^3$ $±4.9x10^3$	$14.8x10^3$ $±1.4x10^3$	<0.0001
Phosphopantetheine (PP)	$97.8x10^3±37.6x10^3$	$3.97x10^3±0.3x10^3$	0.02
CoA-glutathione (CoAG)	$3.813x10^{-4}±6.981x10^{-5}$	$6.11x10^{-6}±1.16x10^{-6}$	<0.001

Supplemental Table 2. Data from metabolomic experiments

The mean ±SEM and *p* values for the metabolites shown in Figure 5. Con, Control; cKO, *Slc5a6*^{cKO}.

	LC_{50} (mg/kg)		Mean dose given (mg/kg/day)	
**Vitamin**	**Human**	**Mouse**	**Human**	**Mouse**
Biotin	10,000	123,000	2.12	45.3
Pantothenic acid	1,443	17,749	25.9	238.7

Supplemental Table 3. Vitamin Doses

The amount of vitamins given to the mice was calculated for the mouse dose equivalent to human dose from Nair & Jacob (2016)(1), with the assumption that mice eat 3g of food and drink 4ml of water each day. The mean human dose was based on five published studies(2-6). LC50 is the concentration that results in 50% mortality (death) in a population.

	8 weeks Normal Diet		8 weeks Plus Vitamins		20 weeks Normal Diet		20 weeks Plus Vitamins	
	Con	cKO	ConV	cKOV	Con	cKO	ConV	cKOV
**No. of Mitochondria**	251	264	240	261	468	530	241	240

**MCI**								
**Minimum**	0.8192	0.8307	0.8681	0.858	0.8111	0.8035	0.8169	0.797
**1Q**	0.9206	0.9612	0.9984	1	0.9751	1.071	1.006	0.9157
**Median**	1.005	1.029	1.077	1.059	1.081	1.457	1.145	1.081
**3Q**	1.177	1.143	1.212	1.139	1.283	2.216	1.287	1.267
**Maximum**	2.24	3.555	2.02	1.639	4.522	7.741	2.737	2.578
**Range**	1.42	2.725	1.152	0.7807	3.71	6.937	1.92	1.781
**Mean**	1.067	1.133	1.129	1.083	1.241	1.848	1.211	1.14
**SD**	0.212	0.3401	0.1924	0.1337	0.4727	1.08	0.3161	0.2901
***p* value**	*p=0.0075* ↑		*p=0.046* ↓		*p<0.0001* ↑		*p=0.013* ↓	

**VOLUME** (μm³)								
**Minimum**	0.0283	0.047	0.0711	0.0526	0.0154	0.0107	0.0492	0.0327
**1Q**	0.4327	0.3779	0.4342	0.5558	0.289	0.2039	0.3973	0.3547
**Median**	0.74	0.6251	0.8492	0.7988	0.4744	0.3562	0.7307	0.6477
**3Q**	1.132	1.018	1.255	1.173	0.7295	0.5963	1.074	1.005
**Maximum**	3.855	3.67	3.889	4.139	3.861	2.581	5.65	2.657
**Range**	3.826	3.623	3.817	4.086	3.845	2.57	5.6	2.624
**Mean**	0.835	0.7909	0.9673	0.9284	0.5892	0.4399	0.8423	0.7189
**SD**	0.5573	0.5977	0.7191	0.5457	0.4757	0.3263	0.7073	0.4487
***p* value**	*p=0.0802 ns*		*p=0.554 ns*		*p<0.0001* ↓		*p=0.1994 ns*	

Supplemental Table 4. Summary of MCI and volume measurements from mitochondrial reconstructions

Cardiac mitochondrial MCI and volume measurements were quantified from 3D reconstructions, from normal diet and vitamin-supplemented control and *Slc5a6*^{cKO} mice at 8 (shown in Supplemental Figure 7) and 20 weeks (shown in Figure 6). ↓, significant decrease in cKO compared to Con, or cKOV compared to ConV; ↑, significant increase in cKO compared to Con; ns = nonsignificant; by unpaired t-test for two sample comparisons. Con, control; cKO, *Slc5a6*^{cKO}; ConV, vitamin-supplemented control; cKOV, vitamin-supplemented *Slc5a6*^{cKO}.

CI	CII	CIII	CIV	CV
MT-ND1	SDHA	UQCRB	COX4I1	ATP5F1A*
MT-ND2	SDHB*	UQCRQ	COX4I2	ATP5F1B
MT-ND3	SDHC	UQCRC1	COX5A	ATP5F1C
MT-ND4	SDHD	UQCRC2*	COX5B	ATP5F1D
MT-ND4L		MT-CYB	COX6A1	ATP5F1E
MT-ND5		CYC1	COX6A2	ATP5MC1
MT-ND6		UQCRFS1	COX6B1	ATP5MC2
NDUFS1		UQCRH	COX6B2	ATP5MC3
NDUFS2		UQCR10	COX6C	ATP5ME
NDUFS3		UQCR11	COX7A1	ATP5MF
NDUFS7			COX7A2	ATP5MG
NDUFS8			COX7B	ATP5MJ
NDUFV1			COX7B2	ATP5MK
NDUFV2			COX7C	MT-ATP6
NDUFAB1			COX8A	MT-ATP8
NDUFA1			COX8C	ATP5PB
NDUFA2			MT-CO1	ATP5PD
NDUFA3			MT-CO2*	ATP5PF
NDUFA5			MT-CO3	ATP5PO
NDUFA6				ATP5IF1
NDUFA7				
NDUFA8				
NDUFA9				
NDUFA10				
NDUFA11				
NDUFA12				
NDUFA13				
NDUFB1				
NDUFB2				
NDUFB3				
NDUFB4				
NDUFB5				
NDUFB6				
NDUFB7				
NDUFB8*				
NDUFB9				
NDUFB10				
NDUFB11				
NDUFC1				
NDUFC2				
NDUFS4				
NDUFS5				
NDUFS6				
NDUFV3				

	down in cKO
	up in cKO
	nonsignificant in cKO
	not in proteomic dataset
*	subunit in qPCR

Supplemental Table 5. Affected subunits of complexes I to V in the electron transport chain in 8-week *Slc5a6*^{cKO} hearts

Gene expression of the different subunits that make up the five complexes of the electron transport chain was analysed in the proteomic data from 8-week control and *Slc5a6*^{cKO} hearts ($q < 0.05$, all fold changes). The majority of the subunits present in the proteomic data were downregulated in the *Slc5a6*^{cKO} hearts: CI 90.2%, CII 100%, CIII 77.8%, CIV 77%, CV 76.5% (highlighted in green) and the non-significant subunits are highlighted in white. The subunits which were not detected in the proteomic data are highlighted in blue. All proteins were downregulated except one complex I subunit, which was upregulated in *Slc5a6*^{cKO} hearts (NDUFS6 highlighted in orange). * denotes the subunit used in the qPCR experiments (Figure 8).

Supplemental Methods

Genotyping of Animals

Mice were genotyped by standard PCR using primers for *Slc5a6* and *Cre*.

**Genotyping Primer**	**Sequence**
Slc5a6LoxP2F	CCTGTCTCTTGAGAAGCCA
Slc5a6LoxP2R	GGCTGTTGGGAAGCTGAGAT
CreF	GCATAACCAGTGAAACAGCATTGCTG
CreR	GGACATGTTCAGGGATCGCCAGGCG

Reverse Transcription PCR (RT-PCR) and Quantitative real-time PCR (qRT-PCR) Primer Sequences

**Primer**	**Sequence**
**RT-PCR**	
Slc5a6Ex6F	ACCATCCTTGGCCCTCAATG
Slc5a6Ex11R	AGAGACAGGCAACGAAGAGC
**qPCR**	
Ndufb8F	TGGGGTGAACCGATACACTG
Ndufb8R	GTAAGGGTACTGCTTCGGACC
SdhbF	CGTTCTCGGCAGAGTCGG
SdhbR	ACCATAGGTCCGCACTTATTCA
Uqcrc2F	CCGGGTCCTTCTCGAGATTTTA
Uqcrc2R	CCGATTCTTGACAGAGGAGCA
Mtco2F	TCAACATGAAACCCCCAGCCA
Mtco2R	GCGGCTAGCACTGGTAGTGA
Atp5a1F	GCCATTTTGTGCCAGTCGTC
Atp5a1R	CATTTTTGGAGACCAGTCCCG
GapdhF	TGTGCAGTGCCAGCCTCGTC
GapdhR	TGACCAGGCGCCCAATACGG
b-actinF	CTGCTCTTTCCCAGACGAGG
b-actinR	AAGGCCACTTATCACCAGCC
NppaF	GAGAGAAAGAAACCAGAGTG
NppaR	GTCTAGCAGGTTCTTGAAATC
Myh6F	CTACTCCTCTTCCTGCCTGTTC
Myh6R	GTCCGTCATTCTGTCACTCAAA

Analysis of mtDNA/nDNA ratio

The method from followed from Quiros et al 2018(7) . Briefly, genomic DNA was extracted from 8 weeks hearts and RNA was removed by RNAase A. qPCR was performed using SYBR green master mix (Thermo Fisher Scientific, in triplicate on a QuantStudio 7 Real-Time PCR System). Primers for mtDNA targets Nd1 and Cytb, and nDNA Hk2 were used. Data were analysed using the comparative Ct method as described(7).

**Primer**	**Sequence**
Hk2F	GCCAGCCTCTCCTGATTTTAGTGT
Hk2R	GGGAACACAAAAGACCTCTTCTGG
Nd1F	CTAGCAGAAACAAACCGGGC
Nd1R	CCGGCTGCGTATTCTACGTT
CytB2F	GGCTACGTCCTTCCATGAGG
CytB2R	AGCGAAGAATCGGGTCAAGG

Quantification of Fibrosis: Image Processing and Analysis Macro

An Ilastik(8) machine learning model was generated using sub-maximum resolution images from the dataset. Partial labelling was applied, and the model was trained until sufficient accuracy was reached (determined visually). The model was trained to distinguish tissue of interest from all other tissues/background.

The ImageJ macro was designed within FIJI(9) (ImageJ, NIH) for open accessibility. Images of maximum resolution and images with 4x reduced maximum linear resolution were extracted and saved separately. The lower resolution images were processed using the trained Ilastik model, with output 8-bit probability maps for tissue probability were exported and binaries using pre-processing of a Gaussian blur (sigma=10 pixels) and an automated global thresholding (Huang algorithm). Binary tissue maps were used to remove all regions of images not applicable for further analysis, along with recording the area of each image containing tissue of interest for analysis. Maximum resolution images were separated into individual colour channels, with the green channel values subtracted from the red channel values (to allow for extraction of pixels that show a red stain vs a yellow negative stain). Whole images were measured for pixel

intensity in the subtracted red channel, using a minimum threshold value 75, only pixels showing a red intensity above this value are included in analysis.

Proteomics

Protein digestion was carried out with S-Trap micro spin columns (Protifi). Protein concentration was determined using BCA protein assay and an equivalent of 50µg was used for digestion. Proteins were reduced with dithiothreitol (DTT) at a final concentration of 20mM (65°C, 30min). Cysteines were alkylated by incubation with iodoacetamide (40mM final concentration, 30min, room temp. in dark) and then acidified by adding 27.5% Phosphoric acid to a final concentration of 2.5%(v/v). The samples were then loaded onto spin columns in 6 volumes of loading buffer (90% methanol 100mM TEAB pH8) and centrifuged at 4000xg for 30s. The columns were then washed with loading buffer (three times) and the flow through discarded. Proteins were digested with trypsin (Worthington) in 50mM TEAB pH8.5, at a ratio of 10:1 protein to trypsin, overnight at 37°C.

Peptides were eluted with three washes of the trap; first 50µl 50mM TEAB, second 50µl 0.1% formic acid and third 50µl 50% acetonitrile with 0.1% formic acid. The solution was frozen then dried in a centrifugal concentrator and reconstituted in 0.1% formic acid 2% Acetonitrile.

Data Independent Acquisition (DIA): 1µl of each peptide sample was loaded per LCMS run. Peptides were separated using an UltiMate 3000 RSLCnano HPLC. Samples were first loaded/desalted onto Acclaim PepMap100 C18 LC Column (5mm Å~ 0.3mm i.d., 5µm, 100Å, Thermo Fisher Scientific) at a flow rate of 10µl min−1 maintained at 45°C and then separated on a 50cm RP-C18 µPAC™ column (PharmaFluidics) using a 60min gradient from 97% A (0.1% FA in 3% DMSO) and 3% B (0.1% FA in 80% ACN 3% DMSO), to 35% B, at a flow rate of 400nl min−1. The eluent was directed to a Thermo Orbitrap Exploris 480 mass spectrometer via Thermo Scientific µPAC compatible EasySpray emitter at a temperature of 290°C, spray voltage 1600V. The total LCMS run time was 90min. Orbitrap full scan resolution was 60,000, RF lens 50%, normalised ACG Target 300%, scan range 390-1600m/z. DIA MSMS were acquired with 49 variable m/z

windows covering 390-1621m/z, at 30000 resolution, dynamic maximum injection time, with ACG target set to 3000%, and normalized collision energy level of 30%.

The acquired data was analysed in DIA-NN version 1.8(10) against mouse proteome database (Uniprot UP000000589-2022.06.06) combined with common Repository of Adventitious Proteins (cRAP), Fragment m/z: 300-1800, enzyme: Trypsin, allowed missed-cleavages: 2, peptide length: 7-30, precursor m/z 300-1800, precursor charge: 1-4, Fixed modifications: carbamidomethylation(C), Variable modifications:Oxidation(M), Acetylation(N-term).

The DIA-NN text output was reformatted using an in-house R script and further processed using Perseus (version 1.6.15) software package.

Comparative proteomic analysis between *Slc5a6*^{cKO} mutants and controls for each experimental group, based on diet (normal diet or vitamin supplementation), was performed to identify differentially expressed proteins in Perseus v2.0.9.0(11). Proteins were filtered for significance (q<0.05) and for fold change (FC) ≤-0.5 and ≥0.5. Pathway analysis was performed using Ingenuity Pathway Analysis (IPA).

References

1. Nair AB, and Jacob S. A simple practice guide for dose conversion between animals and human. J Basic Clin Pharm. 2016;7(2):27-31.
2. Subramanian VS, et al. Mutations in SLC5A6 associated with brain, immune, bone, and intestinal dysfunction in a young child. Hum Genet. 2017;136(2):253-61.
3. Byrne AB, et al. Identification and targeted management of a neurodegenerative disorder caused by biallelic mutations in SLC5A6. NPJ Genom Med. 2019;4:28.
4. Schwantje M, et al. Genetic defect of the sodium-dependent multivitamin transporter: A treatable disease, mimicking biotinidase deficiency. JIMD Rep. 2019;48(1):11-4.
5. Holling T, et al. Novel biallelic variants expand the SLC5A6-related phenotypic spectrum. Eur J Hum Genet. 2022;30(4):439-49.

6. Hauth I, et al. A mild case of sodium-dependent multivitamin transporter (SMVT) deficiency illustrating the importance of treatment response in variant classification. Cold Spring Harb Mol Case Stud. 2022;8(2).
7. Quiros PM, et al. Analysis of mtDNA/nDNA Ratio in Mice. Curr Protoc Mouse Biol. 2017;7(1):47-54.
8. Berg S, et al. ilastik: interactive machine learning for (bio)image analysis. Nat Methods. 2019;16(12):1226-32.
9. Schindelin J, et al. Fiji: an open-source platform for biological-image analysis. Nat Methods. 2012;9(7):676-82.
10. Demichev V, et al. DIA-NN: neural networks and interference correction enable deep proteome coverage in high throughput. Nat Methods. 2020;17(1):41-4.
11. Tyanova S, et al. The Perseus computational platform for comprehensive analysis of (prote)omics data. Nat Methods. 2016;13(9):731-40.
