# Supplementary material for: A Slc5a6-deficient mouse model reveals metabolically driven cardiomyopathy with therapeutic potential for vitamin-based intervention
Source: JCI Insight. 2026 Jun 2;11(14):e200381. doi: 10.1172/jci.insight.200381 (PMC13461167; doi:10.1172/jci.insight.200381)

Uncropped gel in Figure S2A

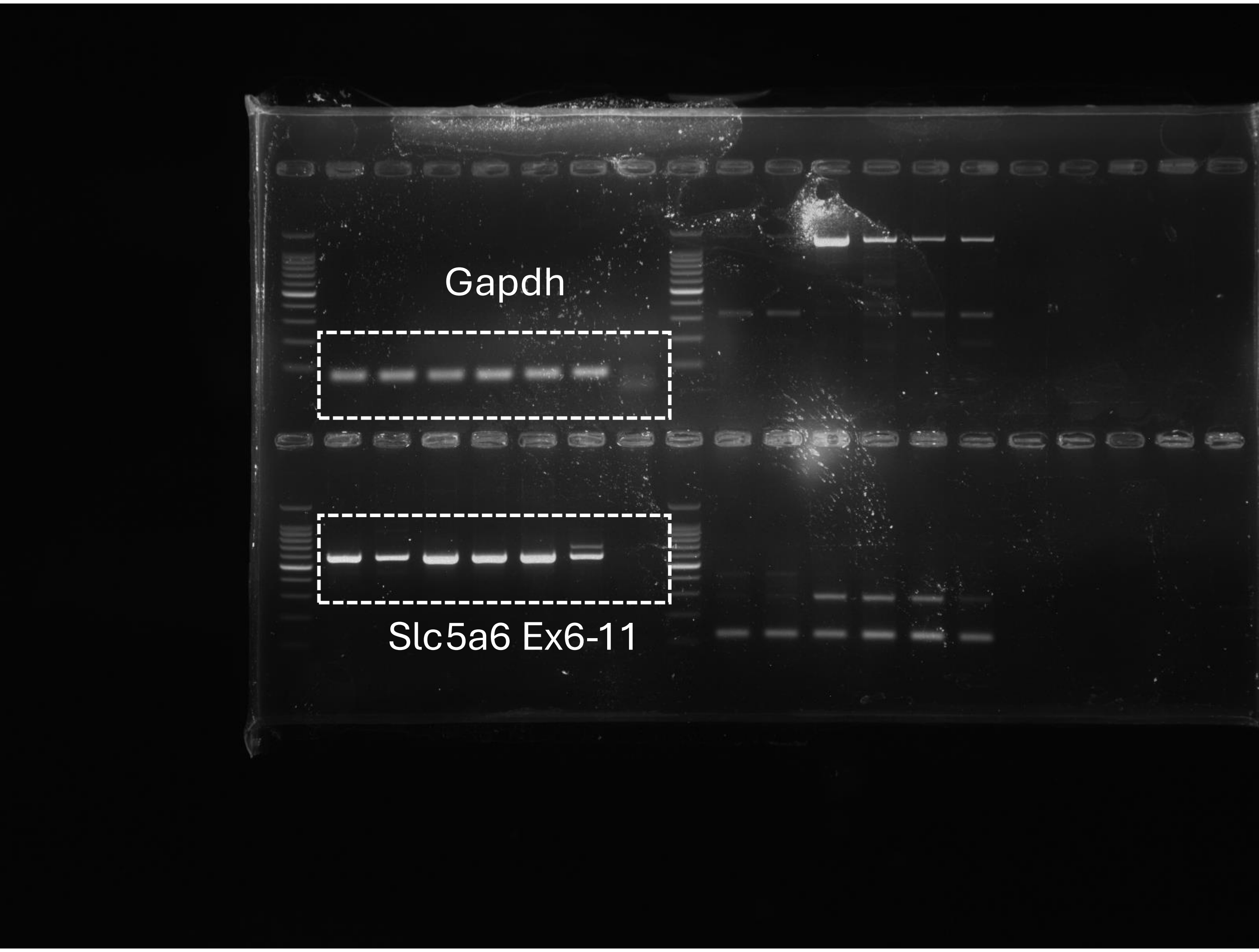

Uncropped gel in Figure S2N

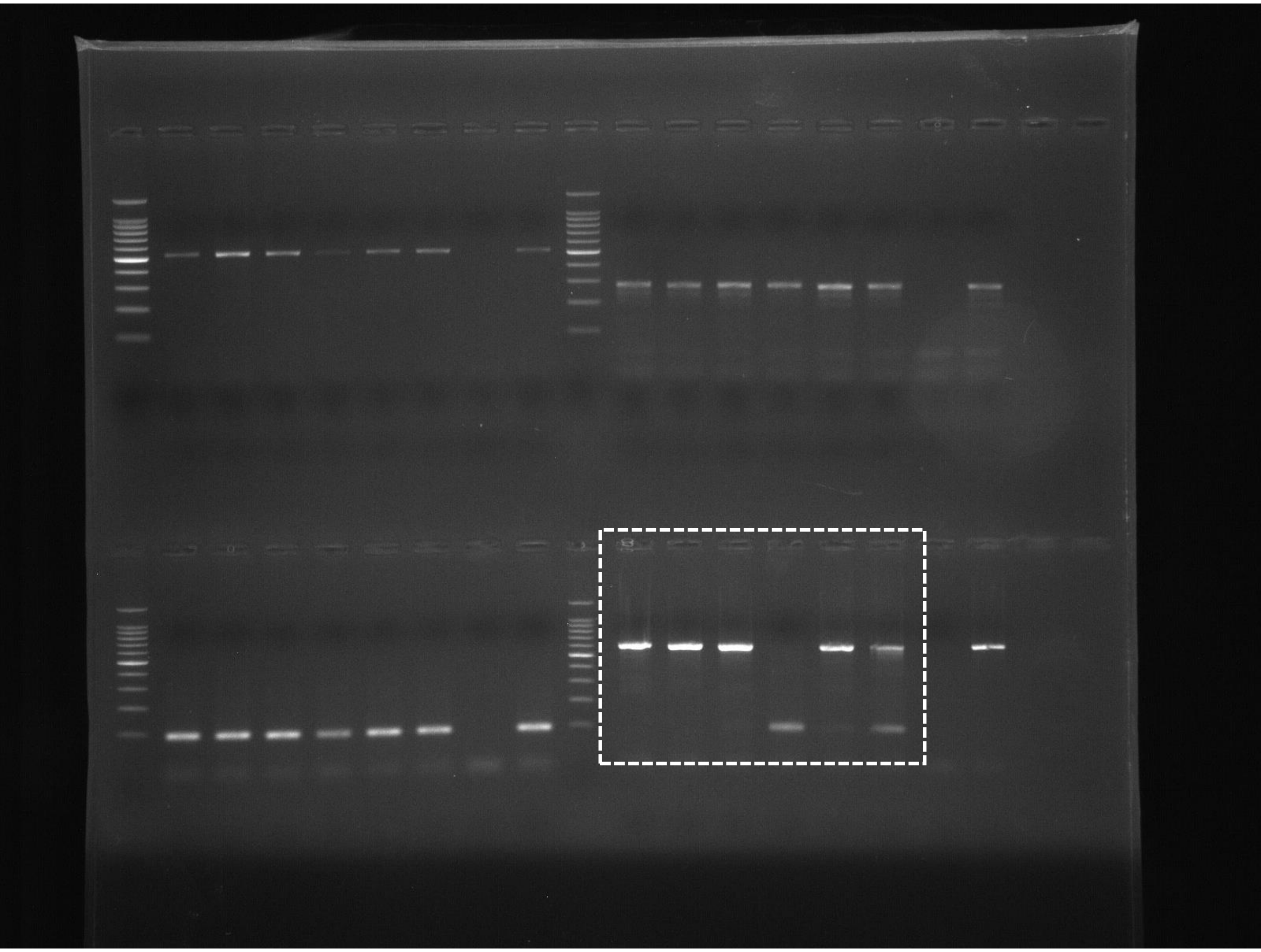

Uncropped blots for Figure S4

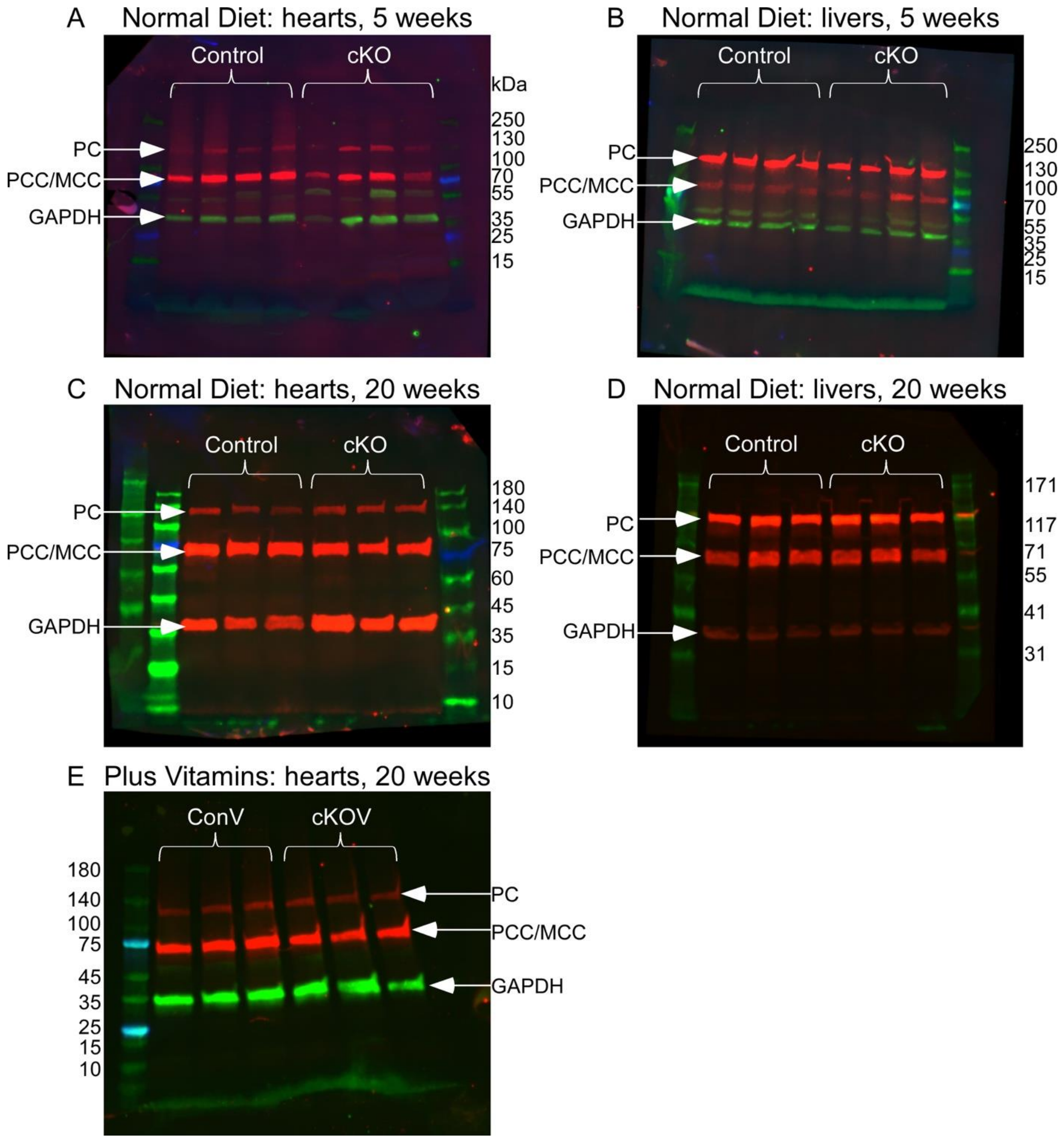

Uncropped blots for Figure S8

A Normal Diet 5 weeks: HADHA

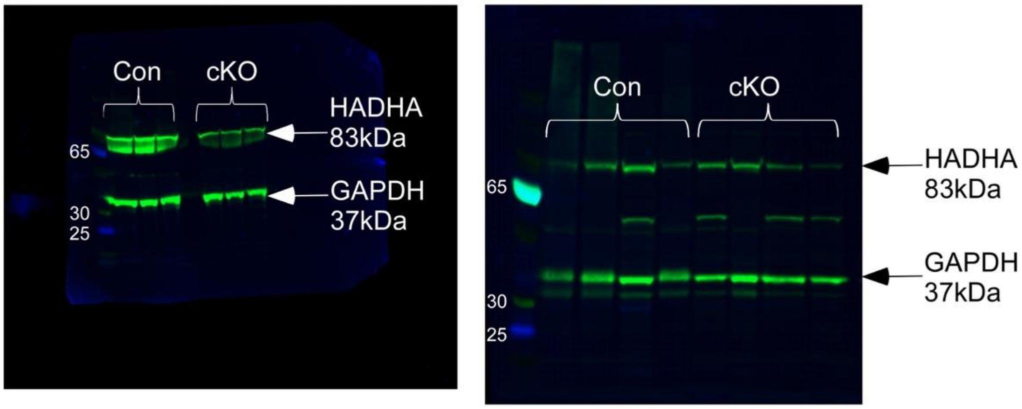

B Normal Diet 5 weeks: MCAD

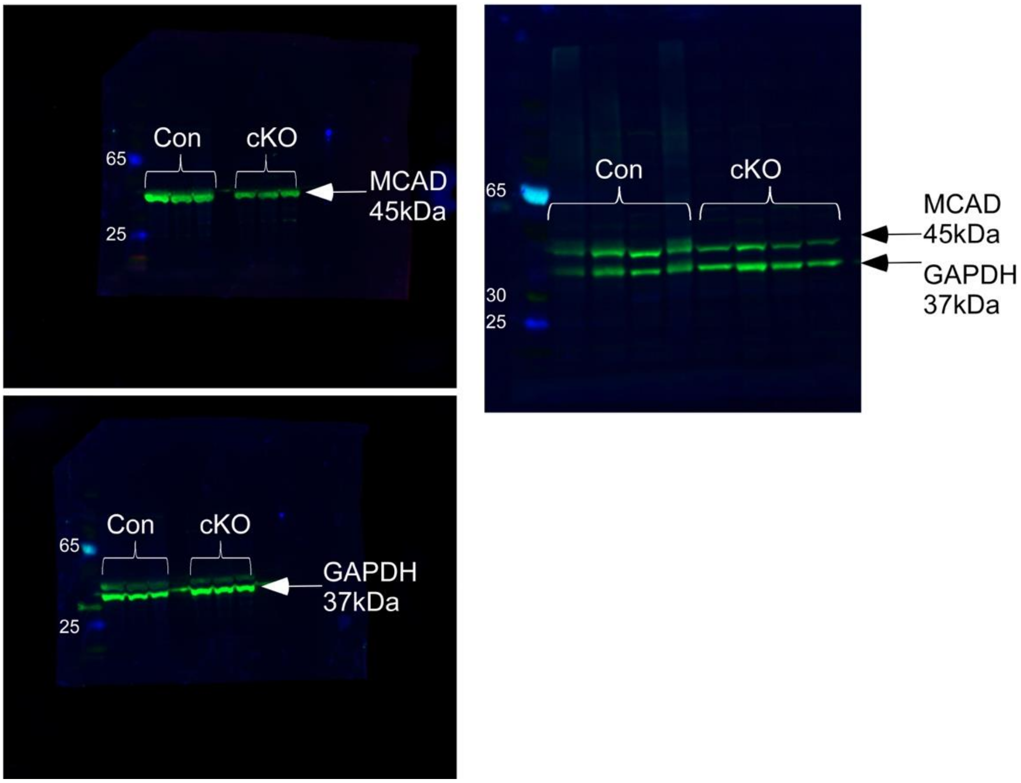

Supplement: Supplementary file 2 [file jciinsight-11-200381-s106.pdf]
